# Spatial Regulation Control of Oxygen Metabolic Consumption in Mouse Brain

**DOI:** 10.1002/advs.202204468

**Published:** 2022-10-18

**Authors:** Lin Zhou, Xinru Li, Bin Su

**Affiliations:** ^1^ Institute of Analytical Chemistry Department of Chemistry Zhejiang University Hangzhou 310058 China

**Keywords:** caffeine, in vivo electrochemistry, neighboring brain regions, oxygen metabolic consumption, spatial regulation control

## Abstract

The mammalian brain relies on significant oxygen metabolic consumption to fulfill energy supply, brain function, and neural activity. In this study, in vivo electrochemistry is combined with physiological and ethological analyses to explore oxygen metabolic consumption in an area of the mouse brain that includes parts of the primary somatosensory cortex, primary motor cortex, hippocampus, and striatum. The oxygen levels at different locations of this boundary section are spatially resolved by measuring the electrical current in vivo using ingeniously designed anti‐biofouling carbon fiber microelectrodes. The characteristics of the current signals are further interpreted by simultaneously recording the physiological responses of the mice. Additionally, ethological tests are performed to validate the correlation between oxygen levels and mouse behavior. It is found that high‐dose caffeine injection can evoke spatial variability in oxygen metabolic consumption between the four neighboring brain regions. It is proposed that the oxygen metabolic consumption in different brain regions is not independent of each other but is subject to spatial regulation control following the rules of “rank of brain region” and “relative distance.” Furthermore, as revealed by in vivo wireless electrochemistry and ethological analysis, mice are at risk of neuronal damage from long‐term intake of high‐dose caffeine.

## Graphical abstract

In vivo electrochemistry is combined with physiological and ethological analyses to explore oxygen metabolic consumption in the mouse brain. The spatial variability of oxygen metabolic consumption evoked by caffeine intake is found to exist between neighboring brain regions. The spatial regulation control of oxygen consumption induces an uneven distribution of oxygen in brain regions and abnormal behavioral changes in mice.

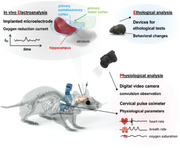

## 1 Introduction

The brain is highly sensitive to oxygen.^[^ ^1^ ^]^ Furthermore, the brain functions heavily rely on the continuous supply and metabolic consumption of oxygen. Brain and neural activity can drive changes in oxygen consumption and uptake from seconds to hours.^[^ ^2^ ^]^ In recent years, diverse technologies, including blood oxygenation level‐dependent functional magnetic resonance imaging (BOLD‐fMRI) and positron emission tomography (PET), have been used to detect brain oxygen metabolic consumption to study brain energy metabolism and its connection with brain function and neural activity.^[^ ^2^, ^3^ ^]^ While global oxygen metabolic consumption can be estimated from BOLD‐fMRI and PET, the result is relatively hysteretic and macroscopic because these techniques have a characteristic temporal resolution of seconds to minutes and spatial resolution of millimeters to centimeters.^[^ ^4^ ^]^ Whether there exists an acceptable spatiotemporal variability in oxygen metabolic consumption in the same or different brain regions remains unclear. Such information will help understand the molecular basis of the coupling between energy metabolism and brain function.

**Scheme 1:**
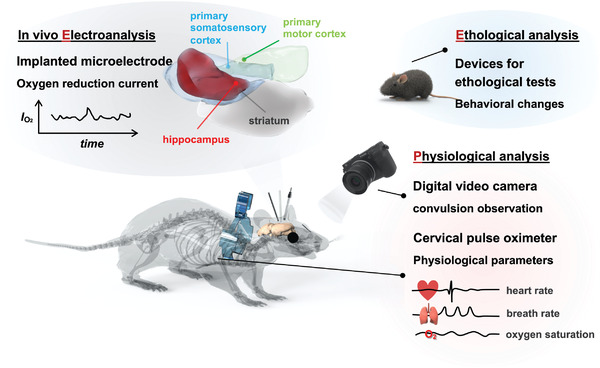
The EPE method that combines in vivo electrochemistry and physiological and ethological analysis for studying the spatial regulation control of oxygen metabolic consumption in the mouse brain.

In this article, we report an approach that combines in vivo electrochemistry with physiological and ethological analyses, designated as the EPE method, to explore the spatial variability of oxygen metabolic consumption in an area of the brain that includes parts of four different brain regions (**Scheme** **1**). A carbon fiber microelectrode (7 µm in diameter)^[^ ^5^ ^]^ modified by a ternary nanoporous membrane was fabricated as a powerful tool to spatially resolve the oxygen level and its variation in different brain regions of live mice. Meanwhile, a cervical physiological sensor and a digital video camera were used to record the physiological responses of mice (such as heart rate, breath rate, arterial oxygen saturation, and convulsion), which allowed us to identify the origin of spatial variability in oxygen metabolic consumption. Furthermore, ethological tests were performed to validate the relevance of oxygen levels to mouse behavior. Using this method and caffeine as the model stimulus, we proposed the existence of spatial regulation control of oxygen metabolic consumption in the brain, including parts of four neighboring brain regions: the primary somatosensory cortex (S1), the primary motor cortex (M1), hippocampus, and striatum. We demonstrate that regulation control is not only driven by neural activity but is also subject to the interaction between brain regions following the rules of “rank of brain region” and “relative distance.” One of the direct consequences of regulatory control is the uneven distribution of oxygen levels in different brain regions, which evokes abnormal behavioral changes in mice. In addition, long‐term in vivo brain oxygen monitoring over weeks enabled by a custom wireless electrochemical system, together with ethological testing, revealed the occurrence of neuronal damage in the brain region after consecutive daily intake of high‐dose caffeine.

## 2 Results and Discussion

### 2.1 Ternary Porous Membrane Modified Microelectrodes for Brain Oxygen Monitoring

**Figure 1:**
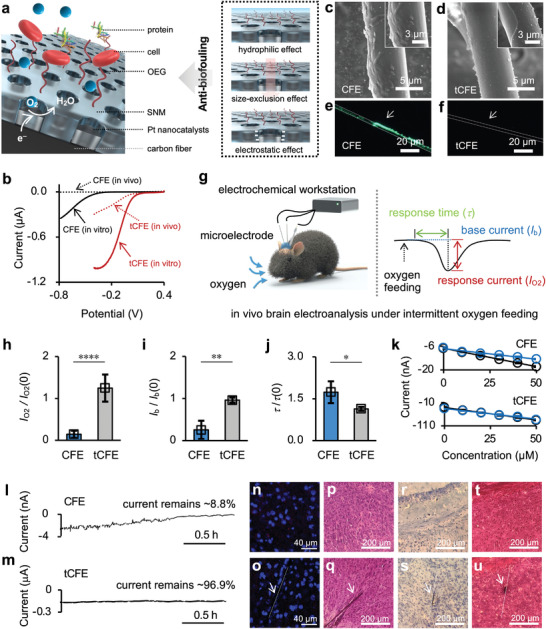
a) Schematic illustration of the structure of tCFE (left) and the anti‐biofouling mechanism (right). b) Linear sweep voltammetry curves (LSVs) obtained with CFE (black) and tCFE (red) in the artificial cerebrospinal fluid (aCSF) (in vitro, solid) and in the hippocampus of the mouse brain (in vivo, dotted). c–f) Scanning electron microscopy (SEM) (c,d) and fluorescence (FL) (e,f) images of CFE and tCFE after being immersed in aCSF containing 20 mg mL^−1^ albumin from bovine serum (BSA) (c,d) or fluorescein 6‐isothiocyanate(isomer II) labeled BSA (FITC‐BSA) (e,f) for 8 h. g) Schematic illustration of the three‐electrode system used for in vivo electroanalysis in the brain of an anesthetized mouse under intermittent pure oxygen feeding (left) and evaluation of the microelectrode performance in terms of response current (*I*_O2_), base current (*I*_b_), and response time (*τ*) (right). h–j) The relative variations of response current (*I*_O2_/*I*_O2_(0), h), base current (*I*_b_/*I*_b_(0), i) and response time (*τ*/*τ*(0), j) of CFE at 2 h and tCFE at 8 h after implantation in the hippocampus in response to pure oxygen feeding (*n* = 3 electrodes). *I*_O2_(0), *I*_b_(0), and *τ*(0) refer to the values measured right after implantation. Data are expressed as mean ± SD. Significance was determined by two‐tailed unpaired Student's *t*‐test (**p < 0.01, ***p < 0.001, and ****p < 0.0001). k) Pre‐calibration (black) and post‐calibration (blue) curves of CFE and tCFE before and after implantation for 8 h in the hippocampus of living mice. Current values were measured in aCSF upon increasing the oxygen concentration successively. Data are expressed as mean ± SD. l,m) Typical chronoamperometric curves of CFE (l) and tCFE (m) implanted in the hippocampus. n–u) Confocal fluorescence images of immunohistochemistry staining (n,o), optical images of H&E (p,q), as well as Nissl (r,s), and Masson's trichrome (t,u) staining of normal brain sections (n,p,r,t) and brain sections with an implanted tCFE (o,q,s,u). The nucleus is shown in blue (DAPI staining) and CD11b protein in red (Cy3 immunohistochemical staining). The inflammatory cells were double stained by DAPI and Cy3. The white dotted line indicates the location of implanted tCFE.

The electrochemical activity, anti‐biofouling ability, and biocompatibility of the microelectrodes were studied to determine the sensitivity, accuracy, and reliability of the current signals measured in the mouse brain. Although carbon fiber microelectrode (CFE) with a diameter of 7 µm is used commonly,^[^ ^6^ ^]^ the severe biofouling of the electrode surface by proteins and cells overwhelmingly limits its applications in continuous and long‐term in vivo electroanalysis.^[^ ^7^ ^]^ To overcome this limitation, we fabricated a new type of CFE modified by a ternary porous membrane structure (designated as tCFE). The ternary structure is composed of a silica nanochannel membrane (SNM), oligopolyethylene glycol (OEG) monolayer grafted on the SNM surface, and platinum (Pt) nanocatalysts buried inside the SNM (**Figure** **1**a and Figures S1–S5, Supporting Information). The SNM consists of highly ordered and closely packed nanochannels with a diameter of ≈2–3 nm and length of ≈160 nm (Figures S3 and S4, Supporting Information), providing a high oxygen permeation flux even after grafting the OEG monolayer on the surface (Figure S6, Supporting Information). Pt nanocatalysts were electrodeposited inside SNM (Figures S2 and S5, Supporting Information), which could not only promote the four‐electron reduction of oxygen,^[^ ^8^ ^]^ but also lower the onset potential of oxygen reduction by ≈0.4 V under both in vitro and in vivo conditions (Figure 1b). In addition, tCFE exhibited a wide linear range and high selectivity for oxygen detection (Figure S2d,e, Supporting Information).

The ternary nanoporous structure offers tCFE an excellent anti‐biofouling ability, arising from the size‐exclusion effect due to ultrasmall and uniform nanochannels of SNM (Figure 1a and Figure S4, Supporting Information), the electrostatic effect due to the negatively charged surface of SNM (a surface charge density of −0.015 C m^−2^),^[^ ^9^ ^]^ and the hydrophilic effect owing to the hydrophilicity of the silica and OEG monolayer (Figure S7, Supporting Information). As shown in Figure 1c–f, and Figures S8 and S9, Supporting Information, proteins and cells are prohibited from adsorbing or adhering to the surface of tCFE, indicating that tCFE possesses excellent anti‐biofouling ability under in vitro conditions. The stability and anti‐biofouling ability of tCFE in the brains of living mice were evaluated using the intermittent pure oxygen feeding model^[^ ^10^ ^]^ and three parameters, namely response current (*I*_O2_), base current (*I*_b_), and response time (*τ*) (as defined in Figure 1g). Feeding pure oxygen to mice resulted in a negative current overshoot (Figure S10a–d, Supporting Information), and tCFE displayed the largest *I*_b_ and *I*_O2_, as well as the smallest *τ*. Moreover, the relative variations of the three parameters, defined as *I*_O2_/*I*_O2_(0), *I*_b_/*I*_b_(0), and *τ*/*τ*(0) (the denominators refer to measured values immediately after implantation), were all insignificant for tCFE at 8 h after implantation (Figure 1h–j and Figure S10e–g, Supporting Information). In addition, the ratio between the slope of the post‐calibration curve and that of the pre‐calibration curve was estimated to be ≈85.7 ± 3.6% for tCFE, which was much larger than that of all other microelectrodes (Figure 1k and Figure S11, Supporting Information), indicating an insignificant loss of analytical sensitivity after being implanted for 8 h, namely, outstanding electrochemical stability. Indeed, the chronoamperometric response of tCFE showed only a slight loss of base current by ≈3.1% after 2 h of in vivo oxygen monitoring, while that of bare CFE was very significant (by ≈91.2%) (Figure 1l,m). These results confirmed that tCFE possesses an excellent anti‐biofouling ability and very stable electrochemical activity in both in vitro and in vivo environments.

On the other hand, we implanted CFE or tCFE in the cerebral cortex of mouse brains for 21 days and used immunohistochemical staining of brain sections to evaluate the biocompatibility of microelectrodes.^[^ ^11^ ^]^ As shown in Figure 1n,o and Figure S12a–e, Supporting Information, no CD11b protein expression was observed around the implanted electrode, which is comparable to the control group of normal sections, indicating that implantation did not induce immunological reaction. It should be noted that cerebral injury can induce severe immunological reactions that result in the expression of CD11b proteins (Figure S12b–e, Supporting Information). Haematoxylin and Eosin (H&E), Nissl, and Masson's trichrome staining tests also revealed that the morphology of the sections and distribution of neural cells around the implanted microelectrode were similar to those in the control group (Figure 1p–u and Figure S12f,g, Supporting Information), while a decrease in neural cell number and scar formation were detected in the injured section (Figure S12i–k, Supporting Information). These results suggested that tCFE is highly biocompatible.

### 2.2 Spatial Regulation Control of Brain Oxygen Metabolic Consumption

Owing to its excellent electrochemical activity, anti‐biofouling ability, and biocompatibility, tCFE is suitable for the long‐term in vivo monitoring of oxygen levels in the mouse brain. Moreover, owing to its small size and high spatial resolution, tCFE can be easily implanted in different locations in the brain to measure the spatial variability of oxygen levels. We further combined in vivo electroanalysis with physiological and ethological analyses (designated as the EPE method, Scheme 1 and Figure S13, Supporting Information) to study the origin and consequence of spatial variability in oxygen metabolic consumption. Caffeine was used as the model stimulus. Overall caffeine intake through common beverages, such as coffee and tea, has prompted extensive studies focusing on their biological effects.^[^ ^12^ ^]^ As an antagonist of all subtypes of adenosine receptors, caffeine acts on neurons and glial cells of the whole brain, affecting brain functions (such as sleep, cognition, and memory) and causing brain dysfunctions and diseases.^[^ ^13^ ^]^

**Figure 2:**
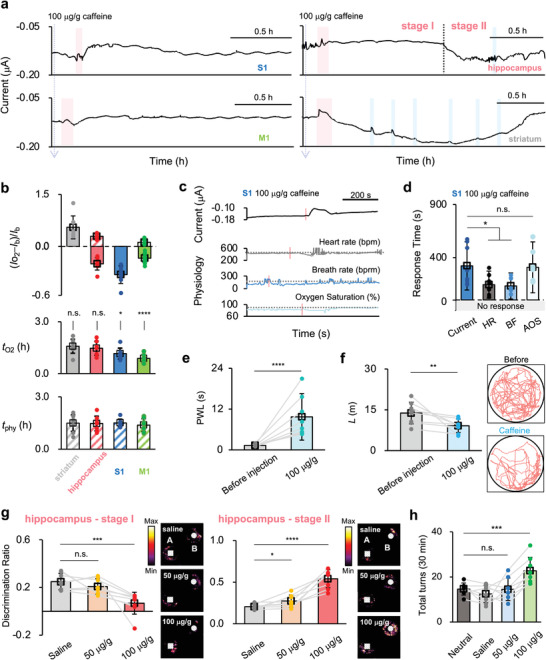
a) Chronoamperometric curves recorded with tCFE in a localized boundary section that included four neighboring brain regions, after high‐dose caffeine injection. The pink and blue bars indicate the occurrence of brain hypoxia and convulsion, respectively. b) Statistical analysis of the variation of the response current relative to the base current ((*I*o_2_ − *I*_b_)/*I*_b_, top), the duration of current variation (*t*o_2_, middle), and the duration of physiological response (*t*_phy_, bottom) in four brain regions. The duration refers to the time required to recover the neutral state level. c,d) Typical chronoamperometric curves recorded in S1 (top) and concurrent physiological responses (bottom) during the brain hypoxia (c) and the corresponding statistical analysis of the response time needed to observe the variations of current, heart rate (HR), breath rate (BR) and arterial oxygen saturation (AOS) (d). The pink lines indicate the moments at which the variations were detected. e) The paw withdrawal latency (PWL) of mice in response to hindpaw pressure stimulation before and after high‐dose caffeine injection (*n* = 8 mice). f) The total distance that mice moved (*L*) before and after high‐dose caffeine injection (*n* = 8 mice). Typical motion tracks of mice in 5 min in an empty circular arena are shown on the right. g) New object recognition (NOR) ability of mice at stage I (left) and stage II (right) of current response in the hippocampus under saline, low‐dose caffeine, and high‐dose caffeine stimulation (*n* = 8 mice). The time that mice spent exploring objects A and B (*t*_A_, *t*_B_) was extracted from the heat maps shown on the right, which was used to calculate the discrimination ratio, namely (*t*_B_−*t*_A_)/(*t*_B_+*t*_A_), to evaluate the NOR ability of mice. h) The total rotations of mice at the neutral state and after saline, low‐dose caffeine, and high‐dose caffeine stimulation (*n* = 8 mice). For (b), (d), and (e–h), data are expressed as mean ± SD. Significance was determined by two‐tailed unpaired Student's *t*‐test (*p < 0.05, **p < 0.01, ***p < 0.001, ****p < 0.0001, and n.s., no significance).

**Figure** **2**a shows the typical chronoamperometric curves recorded with tCFE in four neighboring brain regions: the primary somatosensory cortex (S1), the primary motor cortex (M1), hippocampus, and striatum of mice under high‐dose caffeine (100 µg g^−1^) stimulation. The four brain regions are close to each other (Scheme 1); in this case, we can explore the spatial variability of oxygen metabolic consumption in a small boundary section. The oxygen level in the brain is associated with oxygen metabolic consumption; therefore, the variation in the current recorded by tCFE is closely related to the change in oxygen metabolic consumption in the brain. The measured current variations are analyzed in terms of two parameters (Figure 2b): the relative current variation ((*I*o_2_ − *I*_b_)/*I*_b_, namely the variation in the response current relative to the base current at the neutral state level) and the duration of current variation (*t*o_2_, i.e., the time needed to recover the base current at the neutral state level). In addition, because caffeine intake accelerates the heart and breath rate and elevates arterial blood pressure,^[^ ^13a^ ^]^ physiological parameters, including heart rate, breath rate, and arterial oxygen saturation, were also recorded with a cervical physiological sensor. The duration of the physiological response (*t*_phy_, namely the time needed to recover the neutral state level), which is believed to be closely related to caffeine metabolism, was used as the parameter to interpret the measured current variation further. Typical physiological responses to low‐dose caffeine (50 µg g^−1^) and saline (control group) stimulation are shown in Figure S14, Supporting Information. Figures S15–S18, Supporting Information, indicate the variations in physiological parameters measured at specific stages or times while recording the current in different brain regions.

After a high‐dose caffeine injection, similar positive current overshoots were observed in all four brain regions at ≈1–10 min (as annotated by pink bars in Figure 2a), indicating acute brain hypoxia. After hypoxia, (*I*o_2_ − *I*_b_)/*I*_b_ in S1 and M1 were −0.35 ± 0.11 and −0.14 ± 0.05, with *t*o_2_ of 1.16 ± 0.32 and 0.88 ± 0.23 h, respectively (Figure 2a,b), indicating these two brain regions have a lower oxygen metabolic consumption level than the neutral state. Moreover, *t*o_2_ in S1 and M1 is much shorter than *t*_phy_ (1.46 ± 0.05 h, Figure 2b), indicating that the recovery of oxygen levels in these two brain regions is likely earlier than the complete metabolism of caffeine. This case (i.e., *t*o_2_ < *t*_phy_) is designated as the “stimulus‐unfollowing” behavior. In the hippocampus, the current remained lower than the base current in the first period (0.85 ± 0.25 h) and increased in the second period (0.61 ± 0.26 h). The current variations with time in these two periods are termed stage I and stage II, respectively. The duration of each stage was ≈59 ± 17% and 41 ± 18%, respectively, of the total *t*o_2_ (Figure S19, Supporting Information). Furthermore, the values of (*I*o_2_ − *I*_b_)/*I*_b_ calculated for these two stages were −0.21 ± 0.08 and 0.30 ± 0.06, respectively. In contrast, the current in the striatum after acute hypoxia was always higher than the neutral state level, with (*I*o_2_ − *I*_b_)/*I*_b_ of 0.55 ± 0.33, indicating a higher oxygen metabolic consumption level. Moreover, *t*o_2_ in the hippocampus and striatum was much longer than that in S1 and M1 but approximately equal to *t*_phy_. This implies that the variation in oxygen levels in these two brain regions is almost simultaneous with caffeine metabolism. For the purpose of comparison, we term this case (namely *t*o_2_ = *t*_phy_) as the “stimulus‐following” behavior. Control experiments under low‐dose caffeine and saline stimulation showed that the variation in current was insignificant (Figures S20 and S21, Supporting Information), confirming that the current features in Figure 2a are indeed related to high‐dose caffeine stimulation.

The apparent spatial variability of the oxygen levels in different brain regions merits further investigation. Figure 2c and Figure S22a–c, Supporting Information, illustrate the typical chronoamperometric curves recorded in different brain regions and concurrent physiological responses during hypoxia. The corresponding statistical analysis of the response time, namely the time needed to detect the variation in current or physiological parameters after high‐dose caffeine injection, is summarized in Figure 2d and Figure S22d–f, Supporting Information. Clearly, hypoxia is accompanied by instant inhibition of both heart and breath rates. In addition, arterial oxygen saturation decreased by ≈20% during hypoxia, in agreement with the decrease in current recorded in the four brain regions.

In contrast, hypoxia was not observed after low‐dose caffeine injection, and the arterial oxygen saturation barely changed, although the inhibition of heart rate and breath rate was still evident (Figure S23, Supporting Information). A control experiment showed that saline injection did not induce hypoxia (Figure S24, Supporting Information). These results imply that inhibition of heart rate and breath rate is probably not the dominant cause of brain hypoxia. Global hypoxia most likely originates from the overconsumption of oxygen required for caffeine‐induced systemic excitement, which overwhelms the normal supply level of oxygen and evokes the supplementary oxygen supply from blood vessels to maintain brain functions. Indeed, the simultaneous decrease in arterial oxygen saturation supports this hypothesis.

Neural activity is closely related to the metabolic energy levels of the brain.^[^ ^2^, ^14^ ^]^ The change in neural activity leads to variations in oxygen metabolic consumption, from which the brain can predict the energy demand of future neural activity. We thus proposed that the varying oxygen levels in four different brain regions under high‐dose caffeine stimulation can reflect the excitation or inhibition of brain region functions. To test this hypothesis, we conducted ethological tests and studied the relationship between oxygen levels in different brain regions and brain function. S1 is the brain region controlling the senses of touch, pressure, and pain. Its function was examined using a hindpaw mechanical stimulation test (Figure S25, Supporting Information).^[^ ^15^ ^]^ A constant pressure of ≈20 N was applied to the mouse hindpaw, and the paw withdrawal latency (PWL) in response to the mechanical force was measured. As shown in Figure 2e, the PWL of mice increased remarkably from ≈1.29 ± 0.55 to 9.71 ± 6.83 s after high‐dose caffeine injection, suggesting that mice had an apparent loss of S1 function and thus became insensitive to the stimulation. This behavior coincides with the continuously low level of oxygen in S1 (Figure 2a,b). Second, M1 is one of the major brain regions responsible for the planning and executing motor activities,^[^ ^16^ ^]^ which was evaluated using the locomotor activity test in an empty arena (Figure 2f and Figure S26, Supporting Information). The total distance that the mice moved (*L*) within 5 min after high‐dose caffeine injection decreased by ≈39 ± 17%, which is not that large in comparison with the control group, but was in agreement with a slight decrease in oxygen level and a short *t*_O2_ in M1. Third, the function of the hippocampus, a memory‐related brain region, was studied using a novel object recognition (NOR) test.^[^ ^17^ ^]^ The high‐dose caffeine injection significantly impaired NOR memory (low discrimination ratio) at stage I and enhanced NOR ability (high discrimination ratio) at stage II (Figure 2g and Figure S27, Supporting Information). Finally, striatum function was assessed using the turning behavior test (Figure S28, Supporting Information).^[^ ^18^ ^]^ As shown in Figure 2h, the number of complete rotations recorded after a high‐dose caffeine injection was increased to ≈22 ± 6 per 30 min, indicating a high activity of the striatum, which also matched well with the high oxygen level observed by chronoamperometry in this brain region. Control experiments showed that low‐dose caffeine and saline injections did not affect the behavior of mice, which is consistent with the negligible variance of the current response.

**Figure 3:**
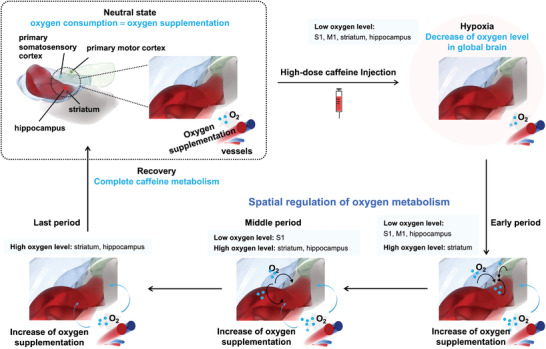
Schematic illustration of spatial regulation control of brain oxygen metabolic consumption in the S1, M1, hippocampus, and striatum under high‐dose caffeine stimulation.

The above ethological tests show that high and low oxygen levels measured by electrochemistry can represent the excitation and inhibition of brain function. The function of the S1, M1, and hippocampus (at stage I) is inhibited, while that of the striatum and hippocampus (at stage II) is excited under high‐dose caffeine stimulation. These results partially differ from recent studies, in which caffeine, as a psychoactive drug, was shown to enhance the neutral activity of all four brain regions with an apparent increase in c‐Fos mRNA expression.^[^ ^19^ ^]^ We propose that the uneven oxygen levels in different brain locations and abnormal behavioral changes in mice most likely originate from the spatial regulation control of oxygen metabolic consumption among the four brain regions. As illustrated in **Figure** **3**, a high‐dose caffeine injection instantly enhances the excitation of the whole brain, which consumes more oxygen than can be supplied, resulting in acute global hypoxia of the brain tissue (Hypoxia in Figure 3). Hypoxia subsequently evokes spatial regulation control of oxygen metabolic consumption among neighboring brain regions. For the periods when the oxygen supply cannot meet the requirement of a high excitement state in the entire brain, oxygen is likely preferentially supplied to the brain region near large blood vessels (the high‐rank brain region) to satisfy the high oxygen metabolic consumption level under high‐dose caffeine stimulation. In contrast, the function of low‐rank brain regions is temporarily inhibited to decrease the oxygen metabolic consumption (Early period in Figure 3). In other words, a high‐rank brain region receives oxygen on priority from nearby low‐rank brain regions to maintain its function and related neural activity, which is designated as the rule of “rank of brain region.” Among the four neighboring brain regions studied, the rank of the striatum was the highest; therefore, a high oxygen consumption level was maintained even in the early period of caffeine metabolism. In contrast, the oxygen levels in the S1, M1, and hippocampus (stage I) decreased, thus inhibiting their functions. Furthermore, among the S1, M1, and hippocampus, the decrease in oxygen consumption level in M1 and the inhibition of motor activity were minor. We ascribe this difference to the rule of “relative distance.” Given that M1 is the brain region most distant from the striatum, both oxygen levels and motor activity decrease only slightly.

With caffeine metabolism, oxygen consumption in the striatum gradually recovers the neutral state level, and mice resume their ability to maintain basic functions of other brain regions (Middle period in Figure 3). The recovery of oxygen consumption levels in the M1 and hippocampus was faster than that in S1. Given that the priority of the hippocampus is higher than that of M1 and S1, and that M1 is the most distant brain region, the oxygen consumption level in the hippocampus is bound to be recovered preferentially, thus accounting for the current increase and enhanced hippocampal memory at stage II. In contrast, the oxygen consumption level in M1 only returned to the neutral state level. The results suggest that the “rank of the brain region” has priority over the “relative distance.” After this period, the hippocampus was no longer affected by the striatum (Last period in Figure 3). During the final stages of caffeine metabolism, the oxygen consumption level in the nearest S1 resumed before recovery of the hippocampus and striatum. Considering that the concentration of caffeine was already shallow during this period, S1 and M1 could not be re‐excited. In this case, the oxygen consumption levels in S1 and M1 were in the neutral state (Neutral state in Figure 3). Overall, the spatial regulation of oxygen metabolic consumption can be interpreted as the “stimulus‐unfollowing” state in S1 and M1, and the “stimulus‐following” state in the hippocampus and striatum.

It should be noted that apart from hypoxia‐induced current decrease, additional positive current overshoots were also observed in the hippocampus and striatum, as indicated by the blue bars in Figure 2a. As interpreted by the physiological responses, they most likely arise from convulsion‐induced respiratory depression. Convulsion inhibits breath and oxygen uptake, thereby instantly decreasing brain oxygen levels. Moreover, similar to brain hypoxia, convulsions were only evoked by high‐dose caffeine injection, and the resulting decrease in oxygen levels was also a global brain crisis. However, due to insufficient oxygen supply, the current variation was hardly recorded in low‐rank S1 and M1 (Figures S29 and S30, and Movie S1, Supporting Information). Control experiments showed that low‐dose caffeine and saline injections did not induce convulsions (Figure S30b, Supporting Information).

### 2.3 Neuronal Damage Induced by Long‐Time Caffeine Intake

**Figure 4:**
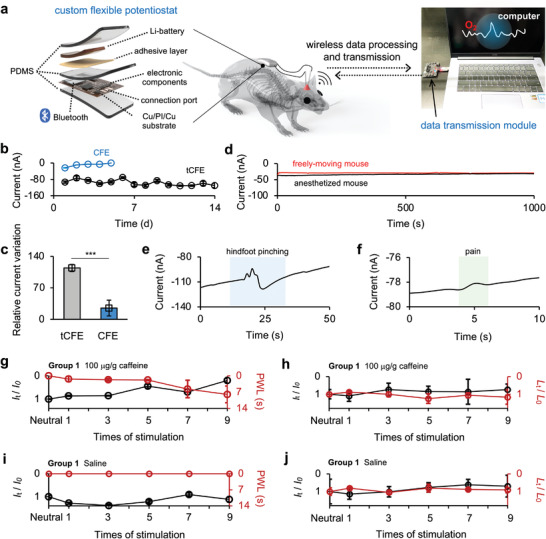
a) Schematic illustration and photograph of the custom wireless electrochemical system for long‐term in vivo electroanalysis on freely‐moving mice, which consists of a custom flexible potentiostat and a wireless data transmission module. b) Comparison of daily measured current recorded with tCFE (black line) and CFE (blue line) implanted in S1 over 14 days using the wireless electrochemical system. Data are expressed as mean ± SD. c) The relative current variation, namely the current measured on the first day with respect to that at the 5th or 14th day after implantation in S1 (*n* = 3 electrodes). Data are expressed as mean ± SD. Significance was determined by two‐tailed unpaired Student's *t*‐test (***p < 0.001). d) Comparison of chronoamperometric curves obtained with tCFE implanted in S1 of freely‐moving (red) and anesthetized (black) mice using the wireless electrochemical system. e,f) Chronoamperometric responses were recorded with tCFE implanted in S1 of a freely‐moving mouse under hindpaw pinching (e) and pain (f) stimulation. g–j) The effect of high‐dose caffeine (g,h) and saline (i,j) on the relative variation of current (*I*_t_/*I*_0_, *I*_0_ refers to the current measured on the day before the experiment and *I*_t_ that measured every 24 h for 9 days) measured with tCFE in S1 (g,i) and M1 (h,j), on the PWL of mice in response to hindpaw mechanical force (g,i) and on the relative variation of total motion distance (*L*_t_/*L*_0_, *L*_0_ refers to the total motion distance measured in the day before experiment and *L*_t_ that measured every 24 h for 9 days). Both the current and behavior of mice were measured daily immediately before caffeine or saline injection. Neutral denotes the day before the experiment. Data are expressed as mean ± SD.

Low levels of oxygen are a well‐known cause of neuronal damage.^[^ ^20^ ^]^ The above experiments clearly show that a low oxygen level in S1 and M1 can last even for several hours under high‐dose caffeine stimulation. Thus, we were curious whether long‐term high‐dose caffeine intake causes neuronal damage. To answer this question, we need to perform both electrochemical measurements of brain oxygen levels on a long‐time scale and ethological tests with freely moving mice. We developed a printed circuit board (PCB)‐based electrochemical system consisting of a custom flexible potentiostat and wireless data transmission module (Figure S31a, Supporting Information). **Figure** **4**a shows the exploded‐view of the potentiostat, including polydimethylsiloxane (PDMS) encapsulation, Li‐battery (45 mAh, 1.0 cm × 1.6 cm × 4.0 mm), adhesive layer, Cu/polyimide(PI)/Cu substrate, and electronic components interconnected by circuit traces. The dimensions and weight of the potentiostat were 2 × 3 cm^2^ and ≈3.5 g, respectively. It was flexible and lean enough to stick to the backs of mice without causing discomfort, and the mice could move freely (Figure S31b,c and Movie S2, Supporting Information). Electrochemical control and data transmission could be executed through Bluetooth communication using the user interface on a personal computer (Figure S31a, Supporting Information).

In the wireless in vivo electroanalysis of freely moving mice, two tCFEs were implanted in the target brain region and wired to the connection ports of the potentiostat on the back of the mouse to monitor the brain oxygen level. Figure 4b shows the current measured daily in S1 by the CFE and tCFE over 5 and 14 days. CFE completely lost its analytical sensitivity after five days because of severe surface biofouling. In contrast, tCFE could persistently record a stable current response for up to 14 days (Figure 4c). This comparison confirms not only the long‐term anti‐biofouling ability of tCFE under in vivo conditions but also the reliable performance of a custom wireless electrochemical system. We then continuously measured the brain oxygen current of the freely moving mouse. Before measurement, the mouse was allowed to rest for over 7 days to recover from the surgery and to acclimatize the implanted tCFE and carry the potentiostat (Figure S32, Supporting Information). As shown in Figure 4d, the chronoamperometric curve recorded for a freely moving mouse is reasonably stable and comparable with that measured for an anesthetized mouse, confirming that the movement of mice does not affect the operation of the wireless potentiostat. In addition, the current sensitivity of the wireless system is also adequate to monitor the variation in brain oxygen levels in mice under hindpaw pinching and pain stimulation (Figure 4e,f).

Using the wireless electrochemical system, the effect of long‐term caffeine intake (high‐dose caffeine injection once per day) on the brain function of mice was investigated by recording the relative variation of current (i.e., *I*_t_/*I*_0_, where *I*_0_ refers to the current measured on the day before the experiment and measured every 24 h immediately before caffeine or saline injection) in S1 and M1 for 9 days. The behaviors of the mice (including PWL in response to hindpaw mechanical force and locomotor activity) were also analyzed (Figure S33a, Supporting Information). We repeated the same experiment using three mice. Figure 4g–j show the results obtained with one group (mouse); those of the other two are shown in Figures S33b–i and S34, Supporting Information. As shown in Figure 4g, the *I*_t_/*I*_0_ recorded in S1 gradually decreased by ≈50% after consecutive daily injections of high‐dose caffeine for 5–7 days. The PWL increased by approximately ninefold (Figure 4h), suggesting a gradual loss of S1 function. Nissl and TUNEL staining confirmed that consecutive high‐dose caffeine injections could cause reduction and apoptosis of nerve cells in S1. This also indicates that the decrease in *I*_t_/*I*_0_ in S1 and the increase in PWL are highly related to neuronal damage (Figures S35 and S36, Supporting Information). In terms of the spatial regulation control network of oxygen metabolic consumption, the inhibitory effect of caffeine on M1 was much weaker than on S1. Indeed, *I*_t_/*I*_0_ in M1 and the relative variation of total motion distance (*L*_t_/*L*_0_, where *L*_0_ refers to the total motion distance measured the day before the experiment and *L*_t_ is measurement captured every 24 h before caffeine injection) of mice were comparable to those in the neutral state (Figure 4i), suggesting that a weak inhibition effect induced by high‐dose caffeine will not cause neuronal damage in M1 (Figures S35 and S36, Supporting Information). Control experiments confirmed that saline injection did not induce neuronal damage in the S1 or M1 (Figure 4i,j and Figures S35 and S36, Supporting Information).

## 3 Conclusion

In summary, we used an approach combining in vivo electroanalysis, as well as physiological and ethological analysis, termed the EPE method, to explore the spatial variability of metabolic oxygen consumption levels in a localized boundary section including the S1, M1, hippocampus, and striatum. A ternary nanoporous structure was ingeniously designed to modify the carbon fiber microelectrode to achieve outstanding electrochemical activity, anti‐biofouling ability, and biocompatibility, which enabled the fabrication of tCFE to work in the brain with insignificant loss of sensitivity for nearly 2 weeks. We demonstrated that the oxygen level varies disproportionately in the localized boundary section of four neighboring brain regions under high‐dose caffeine stimulation, following the rules of “rank of brain regions” and “relative distance.” In other words, there is a spatial regulation control of oxygen consumption between neighboring brain regions. Moreover, the uneven distribution of oxygen levels affects mouse behavior because the activity of brain regions is directly linked to metabolic oxygen consumption and supply. The low oxygen level in the S1, M1, and hippocampus (stage I) accounts for the weak response to hindpaw mechanical stimulation, inhibition of motor activity, and impaired NOR memory in mice. High oxygen levels in the striatum and hippocampus (stage II) indicate improved turning behavior and hippocampal memory. Finally, based on the custom wireless electrochemical measurement and ethological tests, we further revealed that a consecutive low oxygen level induced by long‐term high‐dose caffeine intake would induce neuronal damage in S1, but not M1. This study provides clear evidence that metabolic oxygen consumption in brain regions is not independent of each other but involves cooperative control and mutual interaction.

## 4 Experimental Section

### Materials

All chemicals and reagents were of analytical grade or higher and were used as received without further purification (details are shown in Section S1, Supporting Information).

### Fabrication of tCFE

CFEs were pretreated in 1.0 m NaOH solution by cyclic voltammetry in the potential range of 0–1 V at a scan rate of 0.1 V s^−1^ for ten cycles, and then biased at a constant potential of +1.5 V for 80 s to clean the surface. SNM was then grown on the CFE using electro‐assisted self‐assembly (EASA) approach.^[^ ^21^ ^]^ Subsequently, the OEG monolayer was grafted onto the surface of the SNM via a surface silanization reaction. Finally, the platinum nanocatalysts were electrodeposited inside the SNM by chronoamperometry.^[^ ^22^ ^]^ Further details on the preparation and characterization of tCFE are provided in Supporting Information S2–S4.

### Electrochemical Measurements

Electrochemical measurements were performed using an electrochemical workstation (CHI660D, Chenhua, Shanghai) in a three‐electrode configuration unless otherwise specified. The CFE or modified CFE acted as the working electrode. For in vitro measurements, platinum (Pt) wire, and silver/silver chloride (Ag/AgCl, saturated KCl) were used as the counter and reference electrodes, respectively. In the in vivo electroanalysis, a Pt wire and micro‐Ag/AgCl (aCSF) electrode were positioned outside the mouse cranium to function as the counter and reference electrodes. Chronoamperometry was performed at a constant potential of −0.4 V for tCFE and −0.8 V for others.

### Animals

Male C57 mice (≈20–25 g in weight, 8–10 weeks old) were purchased from the Animal Experimental Center of Zhejiang University (ZJU). All experiments were conducted in accordance with the Guidelines for the Care and Use of Laboratory Animals of ZJU and approved by the Animal Advisory Committee of ZJU (CODE: ZJU20210069). Mice were housed at a constant temperature (≈25 °C) and relative humidity (≈60%) under a 12 h light/dark schedule with food and water ad libitum. The mice were randomly assigned to experimental groups before the experiment. During the data collection, the experimenters were not blinded to the experimental groups or conditions.

### Surgery for Microelectrode Implantation

Mice were anesthetized by injecting pelltobarbitalum natricum (0.1 mg g^−1^) into the abdominal cavity and then mounted in a stereotaxic frame. Using the standard stereotaxic surgical procedure, the microelectrode was implanted in the targeted brain regions, namely striatum (AP = 1.5 mm, L = −0.10 mm, V = 1.7 mm), the primary somatosensory cortex (AP = 1.5 mm, L = −0.34 mm, V = 1.0 mm), the primary motor cortex (AP = 1.0 mm, L = −0.14 mm, V = 1.0 mm), and hippocampus (AP = 1.5 mm, L = −1.5 mm, V = 1.7 mm). The microelectrode, and the counter and reference electrodes can be fixed to the head of mice with dental cement for long‐term studies.

### In Vivo Electrochemical Analyses

Electroanalysis under intermittent pure oxygen feeding was performed as previously reported.^[^ ^10^ ^]^ The microelectrode was implanted into the hippocampus. The pure oxygen atmosphere around the nose of the mice was provided for 10 s every hour. In the in vivo electroanalysis procedure under caffeine stimulation, caffeine solution was injected into the abdominal cavity of anesthetized mice. Current response in the brain was measured using chronoamperometry. The electrode potential was biased at −0.4 V for the tCFE and −0.8 V for the other microelectrodes.

### Physiological Responses

Physiological parameters including heart rate, breath rate, and arterial oxygen saturation, were simultaneously recorded using a clinically validated cervical sensor (MouseOX Plus Pulse Oximeter, Starr). Convulsions were recorded using a digital video camera (RER‐USB13MAF‐V75 USB 2.0, SONY) with a resolution of 3840 × 2160 pixels and a frame rate of 30 FPS. Note that the measurement of the above physiological parameters can be concurrently performed with that of the current.

### Ethological Analysis: Videos

All ethological analyses were based on videos recorded with a digital camera (HIKVISION, China) with a resolution of 1296 × 1080 pixels and a frame rate of 30 FPS.

### Ethological Analysis: Hindpaw Mechanical Stimulation Tests

Mice were handled (1 min per day) for a week to prevent stress. A constant pressure of ≈20 N, controlled by a pressure sensor (Weikesi, China), was applied to the plantar surface of the hindpaw. The PWL of the mice in response to mechanical pressure were determined from the captured videos.

### Ethological Analysis: Locomotor Activity Tests

Mice were handled (1 min per day) to avoid stress and habituated to the empty arena (10 min per day) for a week. The mice were then placed in a circular arena (*d* = 40 cm) to observe their behavior. The total distance moved (*L*) and motion tracks were analyzed using Zootracer software (Microsoft Research, Redmond, Washington, United States).

### Ethological Analysis: NOR Tests

Mice were handled (1 min per day) to avoid stress and habituated to the empty arena (10 min per day) for a week. Objects A and B, with different shapes and colors, were made of wood. Two identical objects (A) were symmetrically placed in an empty arena (*d* = 40 cm). The mice were allowed to explore freely in the arena for 10 min (encoding period). Subsequently, the mice were released into their home cage for some time. The mice were then moved to the arena to explore for 10 min again, where object A was replaced by a new object B (retrieval period). The positions of the objects were counterbalanced to avoid bias. The objects and the arena were thoroughly cleaned with 70% ethanol between the two steps. The time that the mice spent exploring each object during the encoding and retrieval periods was quantified using Zootracer and MATLAB software (MathWorks, Natick, Massachusetts, USA). The discrimination ratio was used as a parameter to evaluate memory activity in mice and hippocampal function.

### Ethological Analysis: Turning Behavior Tests

Mice were handled (1 min per day) for a week to avoid stress. The turning test was then performed in a plastic hemispherical bowl (depth = 15 cm, *d* = 30 cm) with sawdust in the bowl. The mouse was wired with a rotary encoder (E6B2‐CWZ6C, OMRON) and allowed to habituate for ≈10 min. The number of full rotations (360°) in any direction was calculated using MATLAB software.

### In Vivo Electroanalysis of Freely Moving Mice

The wireless electrochemical system consists of a custom miniaturized potentiostat, a data processing module, and a transmission module. A low‐power microcontroller was used to program the required excitation potentials and retrieve readout signals. For chronoamperometry measurements, voltage was applied to the electrode using a digital‐to‐analog converter (DAC). The current was first converted to a voltage signal through a transimpedance amplifier, and then an analog‐to‐digital converter (ADC) was used for voltage signal readout, which was finally transmitted to the user interface on a personal computer through a Bluetooth transceiver. The details of the signal conditioning path for the entire system are provided in the supplementary file. A two‐electrode configuration was used for the wireless in vivo electroanalysis of freely moving mice, in which two tCFEs functioned as the working and counter electrodes. They were implanted using the standard stereotaxic procedure and fixed on the head of the mice using acrylic dentures. The distance between the two tCFEs was controlled to be less than 0.5 mm. After surgery, the mice were allowed to recover for at least one week before the experiments.

### Statistical Analyses

All statistical analyses were performed using Microsoft Excel 2019 (Microsoft Corporation, Redmond, Washington, United States). Data from the failed experimental groups were excluded from the statistical analysis. All data were expressed as the mean ± standard deviation (SD), unless noted otherwise. No statistical methods were used to pre‐determine the sample size, which was annotated in the context or figure captions. For two‐group comparisons, statistical significance was determined using a two‐tailed unpaired Student's *t*‐test. Statistical significance was set at *p <* 0.05. Statistically significant results are indicated in the figures using *p < 0.05, **p < 0.01, ***p < 0.001, ****p < 0.0001, and no significance (n.s., *p* > 0.05).

## Conflict of Interest

The authors declare no conflict of interest.

## Supporting information

Supporting information

Supplemental Movie 1

Supplemental Movie 2

## Data Availability

The data that support the findings of this study are available from the corresponding author upon reasonable request.
