# Supplementary material for: Spatial Regulation Control of Oxygen Metabolic Consumption in Mouse Brain
Source: Adv Sci (Weinh). 2022 Oct 18;9(34):2204468. doi: 10.1002/advs.202204468 (PMC9731700; doi:10.1002/advs.202204468)
Supplement: Supplementary file 1 — Supporting information [file ADVS-9-2204468-s003.pdf]

Supporting Information

Spatial regulation control of oxygen metabolic consumption in mouse brain

*Lin Zhou, Xinru Li and Bin Su**

L. Zhou and X. Li contributed equally to this work

Correspondence and requests for materials should be addressed to B. Su

L. Zhou, X. Li and Prof. B. Su

Institute of Analytical Chemistry, Department of Chemistry, Zhejiang University, Hangzhou 310058, China

E-mail: subin@zju.edu.cn

Table of contents

S1. Chemicals and materials ... 2

S2. Instruments ... 4

S3. Microelectrodes ... 5

S4. In vivo electroanalysis ... 16

S5. Histology and immunohistochemistry ... 20

S6. The EPE system ... 23

S7. Typical physiological responses ... 24

S8. Long-term current variation in the hippocampus ... 30

S9. Responses under low-dose caffeine stimulation ... 31

S10. Caffeine induced brain hypoxia ... 33

S11. Hindpaw mechanical stimulation test ... 36

S12. Locomotor activity test ... 37

S13. New object recognition (NOR) test ... 38

S14. Turning behavior test ... 39

S15. The convulsion ... 40

S16. Neuronal damage induced by long-term caffeine intake ... 42

Movie ... 50

Reference ... 51

S1. Chemicals and materials

Chemicals and reagents. All chemicals and reagents were analytical grade or higher and used as received without further purification. All aqueous solutions were prepared with ultrapure water (18.2 MΩ cm). Cetyltrimethylammonium bromide (CTAB, ≥98%), tetraethyl orthosilicate (TEOS, ≥99.0%), ammonium hydroxide solution (NH_4OH, 28–30%), hexaammineruthenium(III) chloride ($Ru(NH_3)_6Cl_3$, 98%) and Dulbecco’s modified Eagle’s medium (DMEM) were brought from Aldrich. Hydrogen hexachloroplatinate(IV) hexahydrate ($H_2PtCl_6{\cdot}6H_2O$, Pt ≥37.5%), fluorescein 6-isothiocyanate(isomer II) (FITC, 95%), albumin from bovine serum (BSA, 96%), sodium chloride (NaCl, 99.5%), sodium bicarbonate ($NaHCO_3$, ≥99.8%), potassium chloride (KCl, 99.5%), potassium dihydrogen phosphate (KH_2PO_4, 99.5%), disodium hydrogen phosphate dodecahydrate ($Na_2HPO_4{\cdot}12H_2O$, 99%), sodium dihydrogen phosphate dihydrate ($NaH_2PO_4{\cdot}2H_2O$, 98%), sodium sulfate (Na_2SO_4, 99%), magnesium chloride ($MgCl_2$, 99%), calcium chloride ($CaCl_2$, 96.0%), sodium hydroxide (NaOH, 96%), potassium sulfate (K_2SO_4, 99%), caffeine ($C_8H_{10}N_4O_2$, 99%) and paraformaldehyde (PFA, 95%) were purchased from Aladdin. Hydrochloric acid (HCl, 36.0–38.0%) and ethanol (C_2H_6O, 96%) were ordered from Sinopharm. *N*-(Triethoxysilylpropyl)-*O*-polyethylene oxide urethane ($C_{10}H_{22}NO_4SiO(CH_2CH_2O)_{4\text{-}6}H$, OEG-silane, 95%) was obtained from Gelest. Triton X-100 solution (10%), 10× tris-buffered saline (TBS, premixed powder), goat serum and quick antigen retrieval solution for frozen sections, one step TUNEL apoptosis assay kit (C1089) and penicillin-streptomycin solution (PS) were purchased from Beyotime. CD11b antibody (Ab52478) was bought from Abcam. Cy3 goat anti-rabbit IgG (H+L, GB21303), antifade mounting medium with 4’,6-diamidino-2-phenylindole (DAPI), G1006 Masson’s trichrome staining kit and Nissl staining solution were ordered from Servicebio. Hematoxylin (BL702A) and eosin (BL703A) were purchased from Biosharp. 1× Phosphate buffered solution (PBS) and Neg-50 frozen section medium (6502) were purchased from Thermo Fisher Scientific. Denture acrylic was bought from MEYUE. Fetal bovine serum (FBS) was obtained from Gibco.

Artificial cerebrospinal fluid (aCSF). Artificial cerebrospinal fluid (aCSF) was prepared by adding 126 mmol of NaCl, 2.4 mmol of KCl, 0.5 mmol of KH_2PO_4, 27.5 mmol of $NaHCO_3$, 0.5 mmol of Na_2SO_4, 0.85 mmol of $MgCl_2$ and 1.1 mmol of $CaCl_2$ to 1.0 L of ultrapure water.[S1] The pH of aCSF was finally adjusted to 7.2−7.4 by the addition of concentrated HCl and NaOH.

FITC labeled BSA (FITC-BSA). FITC-BSA was prepared by adding 3.75 mL of FITC solution (2 mg mL^{-1}, DMSO) to 5 mL of BSA solution (40 mg mL^{-1}, 10 mM PB, pH = 7.4).[S2] The mixture was then stirred under dark at 4 ^{o}C for 8 h. Finally, 5 mM NH_4Cl aqueous solution was added to quench the reaction. FITC-BSA was stored at 4 ^{o}C under dark for use.

Electrode materials. Carbon fiber (7 μm in diameter) was brought from Toray. Indium tin oxide (ITO) coated glass (surface resistance <15 Ω/square, ITO layer thickness 100 ± 20 nm) was purchased from Kaivo.

S2. Instruments

Main instrument	Manufacturer
CHI660D Electrochemical workstation	Chenhua, China
SU-8020 Field emission scanning electron microscope	Hitachi, Japan
HT770 Transmission electron microscope	Hitachi, Japan
JC2000D2 Contact angle system	Powereach, China
LV100ND Upright optical microscope	Nikon, Japan
68537 Digital stereotaxic system	RWD life science, China
NX50 Freezing microtome	Thermo, Unite states
IX83-FV3000-OSR High-resolution confocal microscope	Olympus, Japan
cx21 Optical microscope	Olympus, Japan
MouseOX plus pulse oximeter	Starr, Unite State
Digital camera	HIKVISION, China
FSR pressure sensor	Weikesi, China
E6B2-CWZ6C Rotary encoder	OMRON, Japan
RER-USB13MAF-V75 USB 2.0 Digital video camera	SONY, Japan

S3. Microelectrodes

Preparation. The preparation of ternary porous membrane modified carbon fiber microelectrode (tCFE) involves three steps (**Figure S1a**).

(1) **Step 1**: silica nanochannel membrane (SNM) was grown on the surface of a bare carbon fiber microelectrode (CFE) by the electro-assisted self-assembly method (**Figure S1b**),[S1,S3] thus obtained microelectrode is designed as CFE_{SNM}. Briefly, a precursor solution was firstly prepared by mixing ethanol with 0.1 M $NaNO_3$ aqueous solution at a volume ratio of 1/1, followed by adjusting pH to 3.0 using concentrated HCl. Subsequently, CTAB and TEOS were added to the mixture at a concentration of 109 mM and 340 mM, respectively, which was further stirred for 2.5 h in an ice-water bath. Then the growth of SNM on the surface of CFE was conducted by chronopotentiometry at a constant current density of –8.0 mA cm^{-2} for 15 s, using a platinum wire and an Ag/AgCl electrode (saturated KCl) as the counter and reference electrodes, respectively. The modified microelectrode was sequentially washed with ultrapure water to remove loosely adsorbed precursors, dried under nitrogen stream and aged at 100 ^{o}C overnight. Finally, CTAB surfactant molecules were excluded from nanochannels of SNM by immersing the electrode in 0.1 M HCl solution for 15 min to obtain CFE_{SNM}.

(2) **Step 2**: OEG monolayer was grafted on the top surface of CFE_{SNM} via surface silanization reaction by immersing CFE_{SNM} in dry toluene containing OEG-silane (0.3 mM) for 18 h at room temperature and thus obtained microelectrode is designed as $CFE_{SNM\text{-}OEG}$.

(3) **Step 3**: electrodeposition of platinum nanocatalysts to nanochannels of $CFE_{SNM\text{-}OEG}$ was carried out by chronopotentiometry in 0.1 M K_2SO_4 containing 0.5 mg mL^{-1} H_2PtCl_6 at a constant potential of –0.2 V and thus obtained electrode is designed as tCFE.[S4] As reported previously, spatially confined electrodeposition of platinum nanocatalysts inside nanochannels of SNM to avoid the overgrowth can be controlled in terms of the characteristics of chronoamperometric curve [S4,S5]. As illustrated in **Figure S2a**, the electrodeposition should be immediately stopped before the current inflexion, otherwise it will overflow to the top surface of $CFE_{SNM\text{-}OEG}$ (the surface morphology of tCFE with a

long electrodeposition time of 250 s is shown in **Figure S2b**). **Figure S2c** displays cyclic voltammograms (CVs) obtained with $CFE_{SNM-OEG}$ and tCFE in 1.0 M H_2SO_4 solution, from which typical redox current features associated with platinum (e.g. adsorption and desorption of hydrogen in the negative potential range) are observed for tCFE.

The linear response range of tCFE for oxygen detection. As shown in **Figure S2d**, the current of oxygen reduction recorded at tCFE increases with increasing the concentration of oxygen in aCSF, yielding a linear range from 0 μM to 1250 μM.

Selectivity against other electroactive substances. Considering other electroactive substances in mice brain, such as ascorbic acid (AA), dopamine (DA), uric acid (UA), 3,4-dihydroxyphenylacetic acid (DOPAC) and 5-hydroxytryptamine (5-HT), might interfere with the reduction of oxygen, the selectivity of tCFE toward oxygen was investigated under the in vitro condition. The electroactive substances were added successively to aCSF and the amperometric current response was recorded continuously (the concentration of these interferents were close to their respective level in mice brain). As shown in **Figure S2e**, no obvious current signals were recorded with the addition of these substances, suggesting the good selectivity of tCFE.

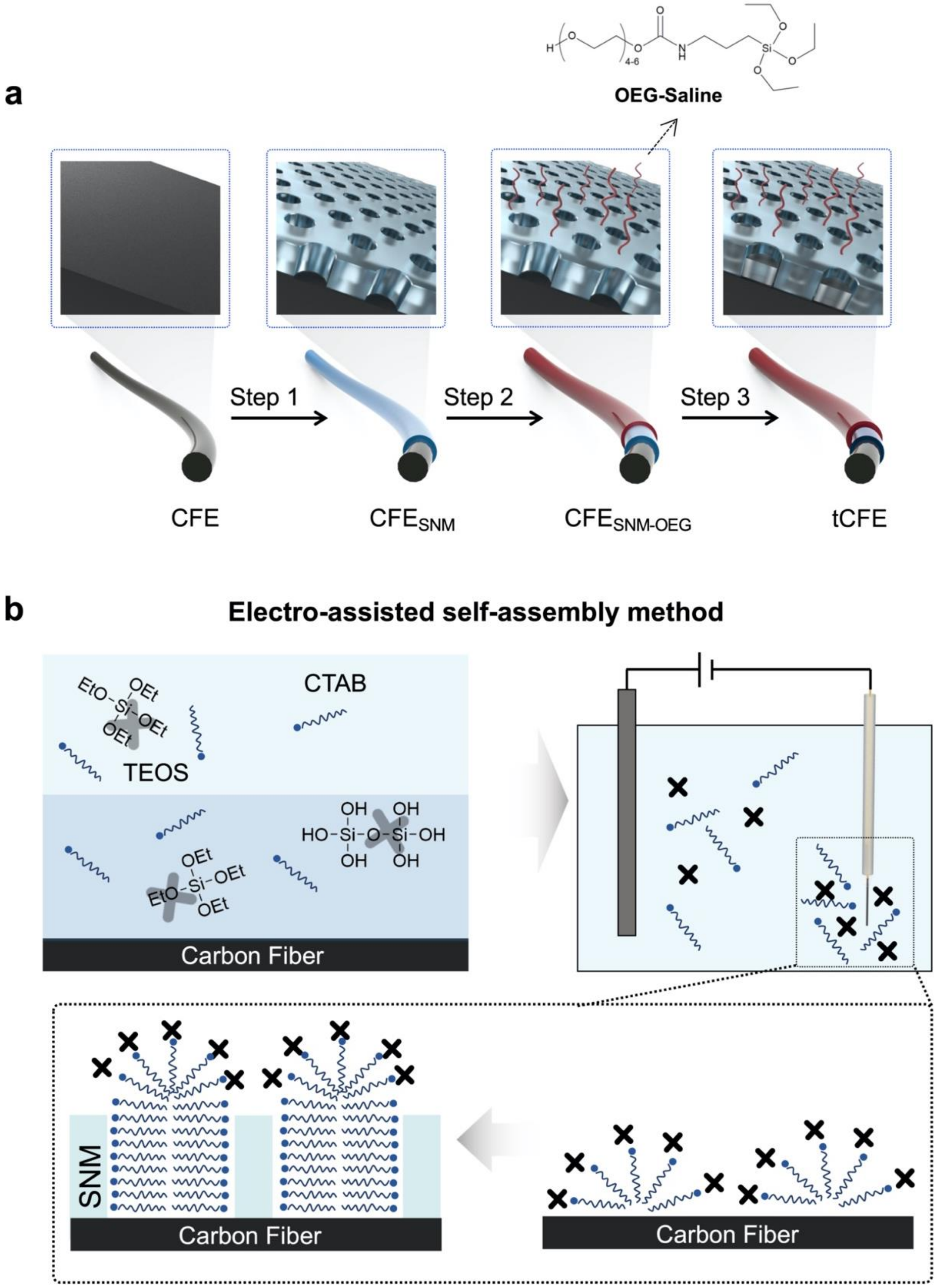

Figure S1. (**a**) Schematic illustration of three steps for tCFE preparation. (**b**) Schematic illustration of growing SNM on CFE by the electro-assisted self-assembly method.

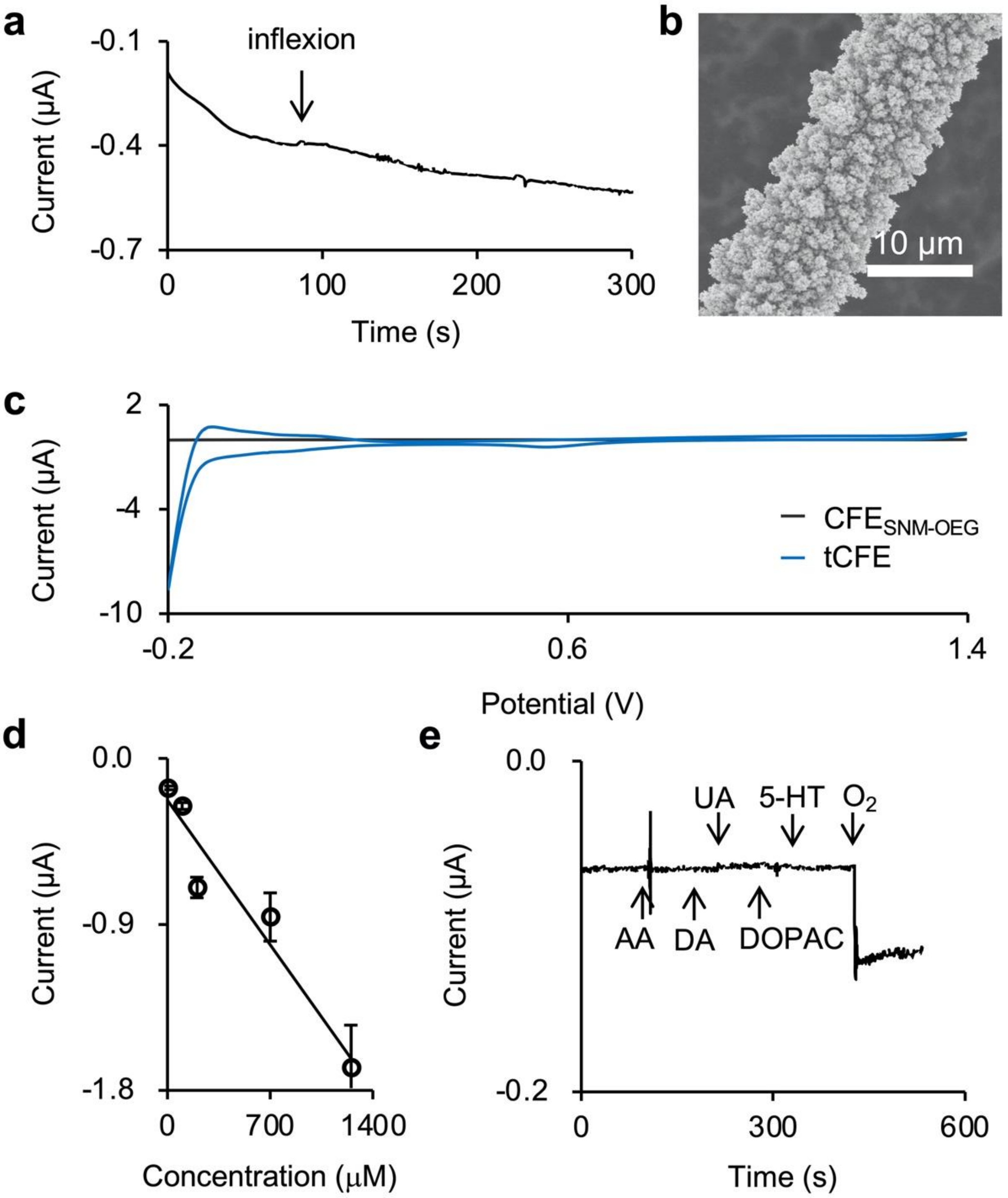

Figure S2. (**a**) Chronoamperometric curve recorded during the electrodeposition of platinum nanocatalysts onto tCFE at a constant potential of –0.2 V. The plating solution was 0.1 M K_2SO_4 containing 0.5 mg mL^{-1} H_2PtCl_6. A platinum wire and an Ag/AgCl (saturated KCl) were used as the counter and reference electrodes, respectively. (**b**) Scanning electron microscopy (SEM) image of tCFE with a platinum electrodeposition time of 250 s. (**c**) CVs obtained with $CFE_{SNM\text{-}OEG}$ (black) and tCFE (blue) in 1.0 M H_2SO_4. The scan rate was 100 mV s^{-1}. (**d**) The linear response range of tCFE for oxygen detection in the concentration range of 0 – 1250 μM. The electrode potential was biased at –0.4 V. (**e**) Amperometric response curve obtained with tCFE upon successive addition of 200 μM AA, 50 μM DA, 50 μM UA, 50 μM DOPAC, 50 μM 5-HT and 50 μM O_2 to aCSF. The electrode potential was biased at –0.4 V.

Structure and morphology characterization. Supplementary **Figures 3** and **4** show the morphology and structure of CFE, CFE_{SNM}, $CFE_{SNM\text{-}OEG}$ and tCFE characterized by scanning electron microscope (SEM, SU-8020 field emission scanning electron microscope, Hitachi) and transmission electron microscope (TEM, HT770 transmission electron microscope, Hitachi). As can be seen from SEM images in **Figure S3a–b**, SNM is compact and covers completely the surface of CFE. The surface of $CFE_{SNM\text{-}OEG}$ remains smooth and no silane aggregates are formed (**Figure S3c**). In the case of tCFE, no platinum nanoparticles are found on the surface (**Figure S3d**), proving they are spatially confined inside nanochannels of SNM. The top-view and cross-sectional TEM images in **Figure S4a–b** show that SNM consists of highly ordered and closely packed nanopores with a diameter of 2–3 nm and a porosity of ~34%, and that the thickness of SNM is ~160 nm. After grafting the OEG monolayer on the top surface of SNM, the nanopore size and the thickness of SNM are comparable to those of pristine SNM (**Figure S4c–d**), indicating the modification of OEG monolayer does not block nanopores. Energy dispersive x-ray spectroscopy (EDX) analysis also demonstrates that carbon (C), oxygen (O), silicon (Si) and platinum (Pt) elements are uniformly distributed on tCFE (**Figure S5**).

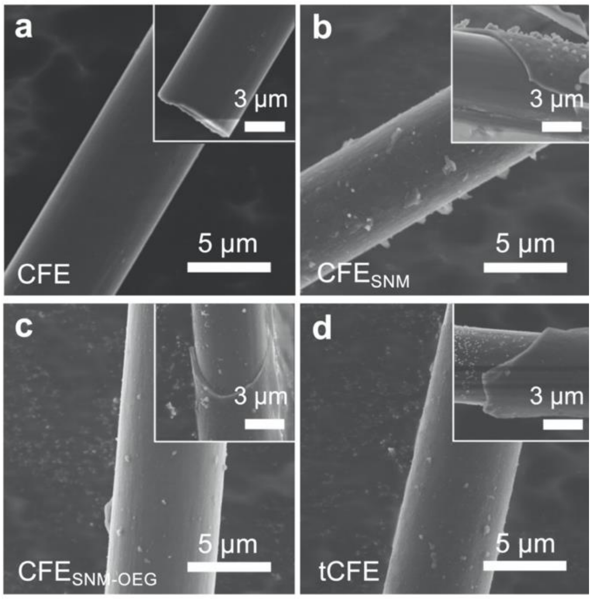

Figure S3. SEM images of CFE (**a**), CFE_{SNM} (**b**), $CFE_{SNM\text{-}OEG}$ (**c**) and tCFE (**d**). The insets show the fracture boundary to prove the formation of ternary membrane layer.

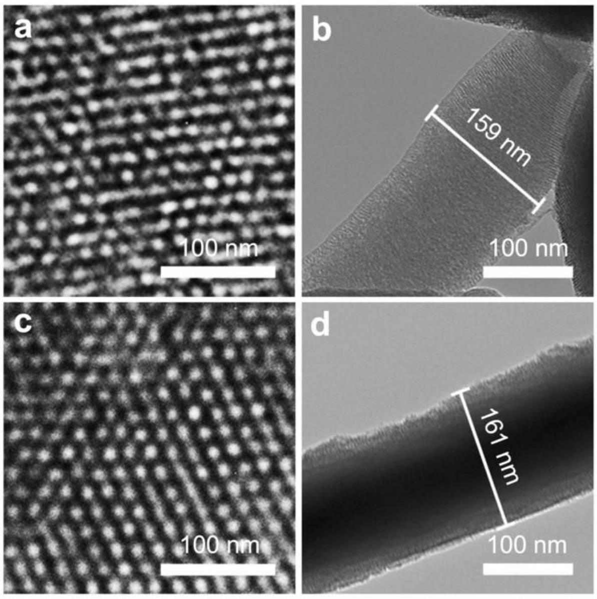

Figure S4. TEM images showing the top surface (**a, c**) and cross-section (**b, d**) of SNM before (**a, b**) and after (**c, d**) grafting the OEG monolayer on the top surface.

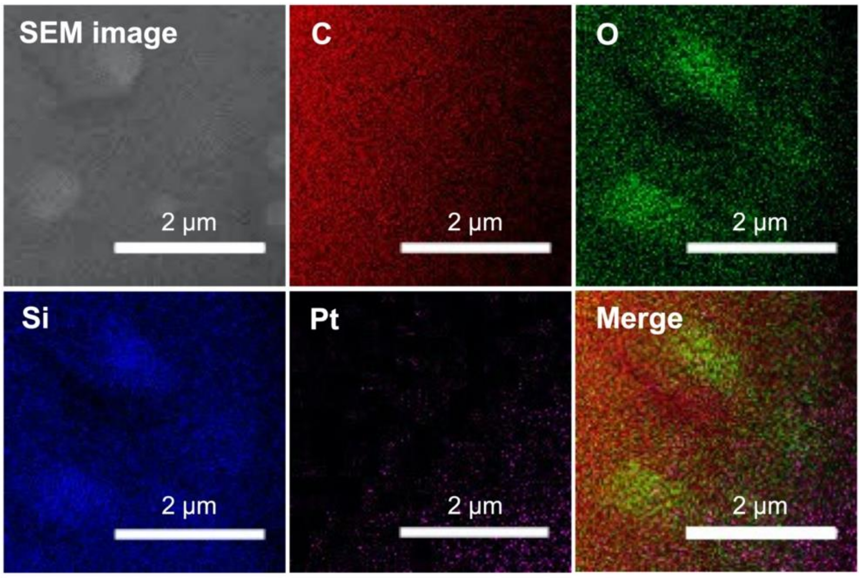

Figure S5. SEM image and EDX analysis of different elements on tCFE.

Molecular permeation characterization. The molecular permeability of SNM and OEG monolayer was investigated by voltammetry measurements, using $Ru(NH_3)_6^{3+}$ as the redox probe. As shown in **Figure S6**, the magnitude of steady-state current plateau of $Ru(NH_3)_6^{3+}$ obtained with CFE_{SNM} is ~2 fold larger than that of bare CFE, because of a high porosity and

a negatively charged surface of SNM. The negatively charged surface of SNM arises from the deprotonation of surface silanol groups.[S1] Moreover, the magnitude of steady-state current plateau obtained with $CFE_{SNM\text{-}OEG}$ is comparable to that of CFE_{SNM}, indicative of the formation of loosely bound monolayer of OEG monolayer and its high molecular permeability.

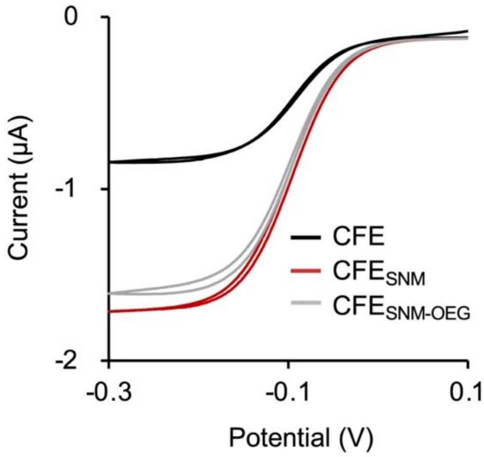

Figure S6. CVs obtained with CFE (black), CFE_{SNM} (red) and $CFE_{SNM\text{-}OEG}$ (grey) in 0.1 M KCl containing 5 mM of $Ru(NH_3)_6^{3+}$. A platinum wire and an Ag/AgCl electrode (saturated KCl) were used as the counter and reference electrodes, respectively. The potential scan rate was 20 mV s^{-1}.

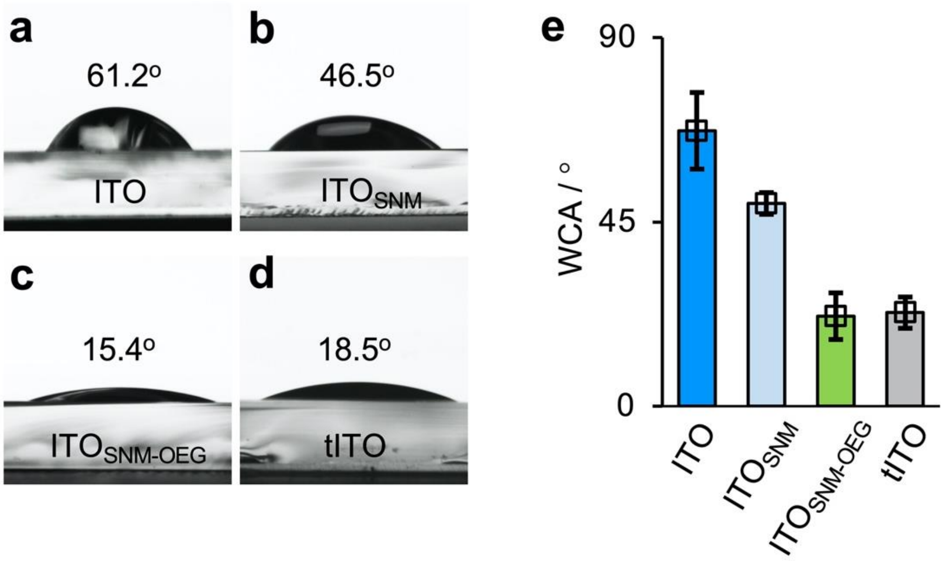

Figure S7. Photographs of water contact angle (WCA) measurements (**a**-**d**) and the values of WCA (**e**) of ITO, ITO_{SNM}, $ITO_{SNM\text{-}OEG}$, and tITO electrode surfaces ($n = 3$ electrodes). Data are expressed as mean ± s.d.

Hydrophilic characterization. Non-specific adsorption of proteins and adhesion of cells tend to occur on hydrophobic surfaces.[S6-8] So the hydrophilicity is often used as a parameter to evaluate the anti-biofouling ability of solid surfaces. Because the surface area of CFE (*d* = 7 mm) is pretty limited, the hydrophilicity characterization based on the water contact angle (WCA) measurement (JC2000D2 contact angle system, Powereach) was conducted with large-area ITO electrodes. SNM was grown on the ITO electrode surface using the Stöber-solution growth approach.[S9] Briefly, the precursor solution was firstly prepared by adding 30 mL of ethanol, 160 mg of CTAB, 10 μL of ammonia aqueous solution and 80 μL of TEOS to 70 mL of ultrapure water under stirring. Then, the ITO electrode was immersed in the precursor solution and SNM was grown chemically at 60 ^{o}C for 12 h. As-prepared electrode was sequentially washed with ultrapure water to remove loosely bounded precursors, dried under nitrogen stream and aged at 100 ^{o}C overnight. After excluding CTAB surfactant molecules from nanochannels of SNM in 0.1 M HCl ethanol solution for 15 min, the ITO electrode modified by SNM (namely ITO_{SNM}) was obtained. Further grafting the OEG monolayer on the top surface of SNM and electrodepositing platinum nanocatalysts inside nanochannels of SNM, using the same protocols as carbon fiber microelectrode, $ITO_{SNM\text{-}OEG}$ and tITO were fabricated. **Figure S7** shows the water contact angle (WCA) measurements and the angels measured for ITO, ITO_{SNM}, $ITO_{SNM\text{-}OEG}$ and tITO surfaces. Apparently, the WCA of $ITO_{SNM\text{-}OEG}$ and tITO decreases to ~21.9 ± 3.3^{o} and ~22.8 ± 2.2^{o}, confirming that the OEG monolayer can indeed offer a pretty hydrophilic surface.

In vitro anti-biofouling ability. Figure S8a-b compares the surface morphology of CFE_{SNM} and $CFE_{SNM\text{-}OEG}$ after being immersed in aCSF containing 20 mg mL^{-1} BSA (pH = 7.2–7.4) for 8 h. Similar to the bare CFE (**Figure 1c** in the manuscript), the adsorption of proteins can be observed on the surface of CFE_{SNM} (**Figure S8a**), suggesting the limited anti-biofouling ability of SNM. In contrast, the adsorption of BSA on the surface of $CFE_{SNM\text{-}OEG}$ (**Figure S8b**) and tCFE (**Figure 1d** in the manuscript) is effectively prohibited, indicating the surface modified with the OEG monolayer possesses an excellent anti-biofouling ability. The anti-biofouling ability of $CFE_{SNM\text{-}OEG}$ and tCFE was also confirmed by fluorescence imaging,

using FITC-BSA as the biofouling reagent (**Figure 1e–f** in the manuscript and **Figure S8c−d**).

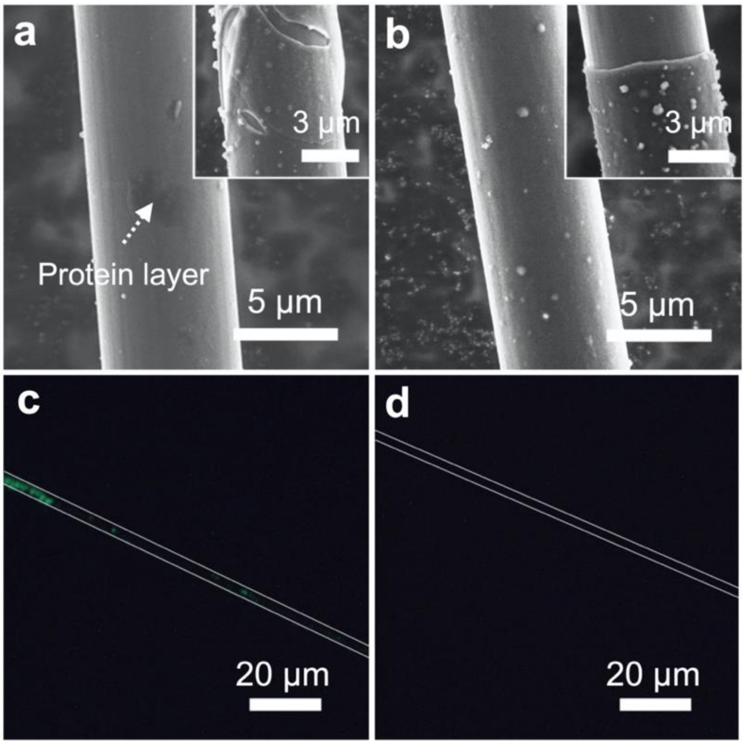

Figure S8. SEM (**a**, **b**) and fluorescence (**c**, **d**) images of CFE_{SNM} (**a**, **c**) and $CFE_{SNM\text{-}OEG}$ (**b**, **d**) after immersing in aCSF containing 20 mg mL^{-1} BSA (**a**, **b**) and FITC-BSA (**c**, **d**) for 8 h. The fluorescence images were captured with an upright optical microscope (LV100ND, Nikon).

Anti-cell adhesion characteristics. Hela cells obtained from the cell bank of Chinese Academy of Sciences were cultured in DMEM supplemented with 10% FBS and 1% PS. Cultures were incubated in cell culture dishes at 37 °C in 5% CO_2 atmosphere, and the growth medium was exchanged every two days. To examine the anti-cell adhesion performance, large-area ITO electrodes were also used, because the surface area of CFE (d = 7 mm) is pretty limited. Hela cells were trypsinized, plated on ITO, ITO_{SNM}, $ITO_{SNM\text{-}OEG}$ and tITO electrodes and incubated for 12 h (**Figure S9a**). Finally, the culture medium was discarded and cells were fixed with paraformaldehyde for 5 min. Because of poor anti-biofouling ability of ITO and limited anti-biofouling ability of ITO_{SNM}, the adhesion of Hela cells can be

observed on the surface. The surface coverage of cells on ITO and ITO_{SNM} was 8.2 ± 3.9% and 5.4 ± 4.3%, respectively. In contrast, unwanted cell adhesion was not observed on the surface of $CFE_{SNM-OEG}$ and tCFE, and the surface coverage of cells was lower than ~2%, indicating the surface modified with OEG monolayer has an excellent anti-cell adhesion characteristic (**Figure S9b–f**).

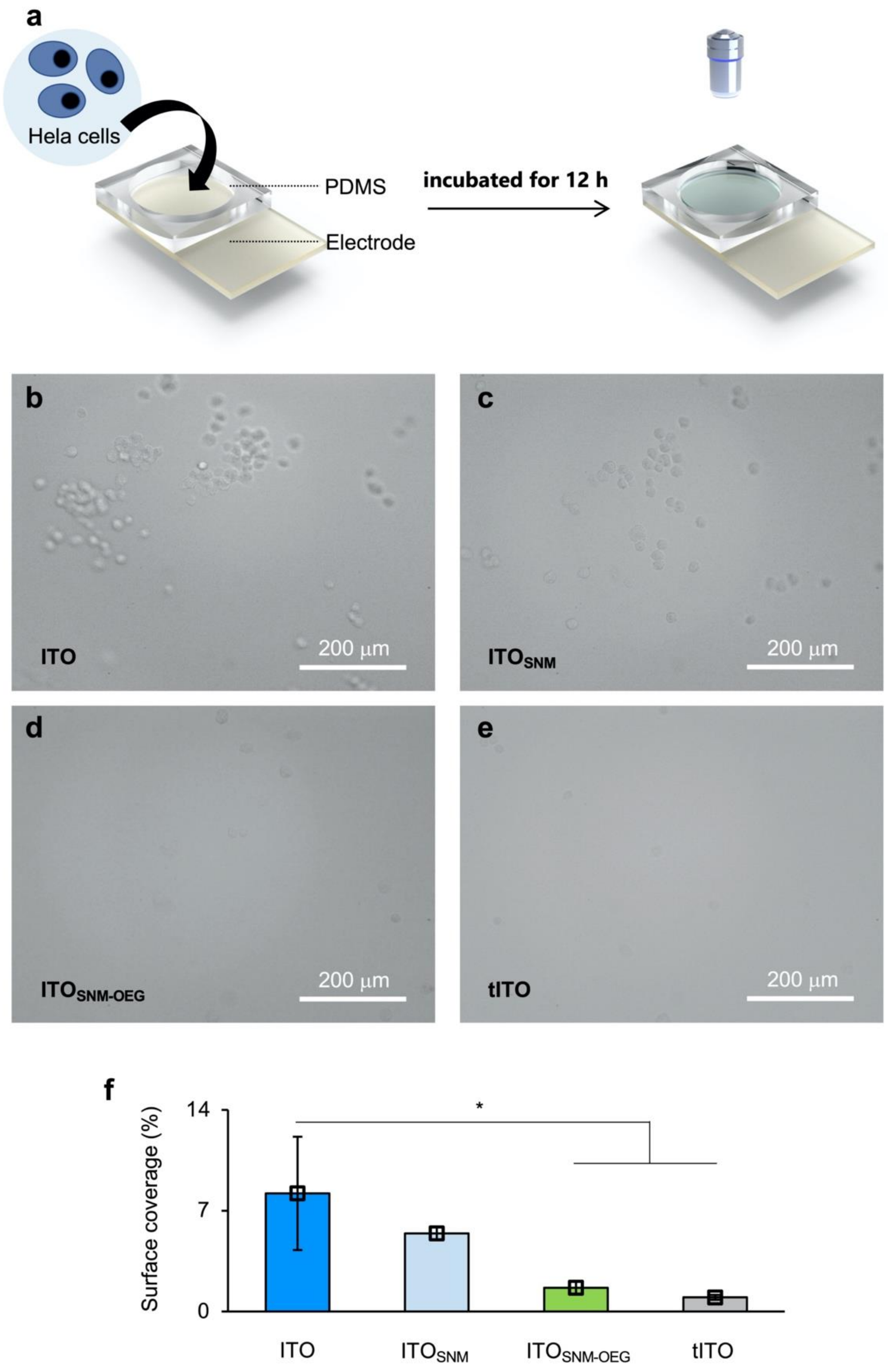

Figure S9. (**a**) Schematic illustration of cell culture on the surface of electrode. (**b**–**e**) Optical images showing the adhesion of Hela cells on ITO (**b**), ITO_{SNM} (**c**), $ITO_{SNM\text{-}OEG}$ (**d**) and tCFE (**e**). (**f**) Statistical analysis of the surface coverage of Hela cells on various electrodes (n = 3 electrodes). Data are expressed as mean ± s.d. Significance was determined by two-tailed unpaired Student's t-test (*p < 0.05).

S4. In vivo electroanalysis

In vivo electroanalysis under pure oxygen feeding. Figure S10a–d compares typical chronoamperometric curves of CFE, CFE_{SNM}, $CFE_{SNM\text{-}OEG}$ and tCFE implanted in the hippocampus of mouse brain in response to pure nitrogen feeding (control) or intermittent pure oxygen feeding. In the latter case, the feeding resulted in a negative current overshoot, but severe surface biofouling caused a remarkable loss of electrochemical activity over time, in particular for the bare CFE. **Figure S10a** shows the chronoamperometric curves recorded with CFE implanted in the hippocampus of mouse brain in response to pure oxygen feeding for 8 h. Because of poor anti-biofouling ability of CFE, the response current (I_{O2}) and response time (τ) (see definitions in **Figure 1g** of the manuscript) in response to intermittent oxygen feeding completely diminished at 2 h after implantation. Meanwhile, the base current (I_b) also decreased by ~75 ± 13% (**Figure 1h–j** in the manuscript). The SNM can slightly enhance the anti-biofouling performance of microelectrode. As shown in **Figure S10b**, the response of CFE_{SNM} remained relatively stable up to 4 h, with I_{O2} and I_b decreased by ~70 ± 8% and ~36 ± 26%, respectively. And τ is increased from ~15.2 ± 3.3 s to ~27.6 ± 4.7 s (**Figure S10e–g**). It is implied that the stability and anti-biofouling ability of CFE_{SNM} are still insufficient for long-term in vivo electroanalysis. In contrast, the electrochemical activity of $CFE_{SNM\text{-}OEG}$ is much better, none of I_{O2}, I_b and τ varies significantly at 8 h after implantation (**Figure S10c**, **e–g**). In comparison with $CFE_{SNM\text{-}OEG}$, thanks to the catalytic effect of Pt nanocatalysts, tCFE illustrates the shortest response time, the largest response current and base current, all of which vary insignificantly at 8 h after implantation. (**Figure S10d** and **Figure 1h–j** in the manuscript).

In order to prove that the current response is indeed related to the variation of brain oxygen level induced by the pure oxygen feeding, the mice were also fed with pure nitrogen. In this case, a positive current overshoot is observed due to the decrease of brain oxygen level (the most left graphs in **Figure S10a–d**).

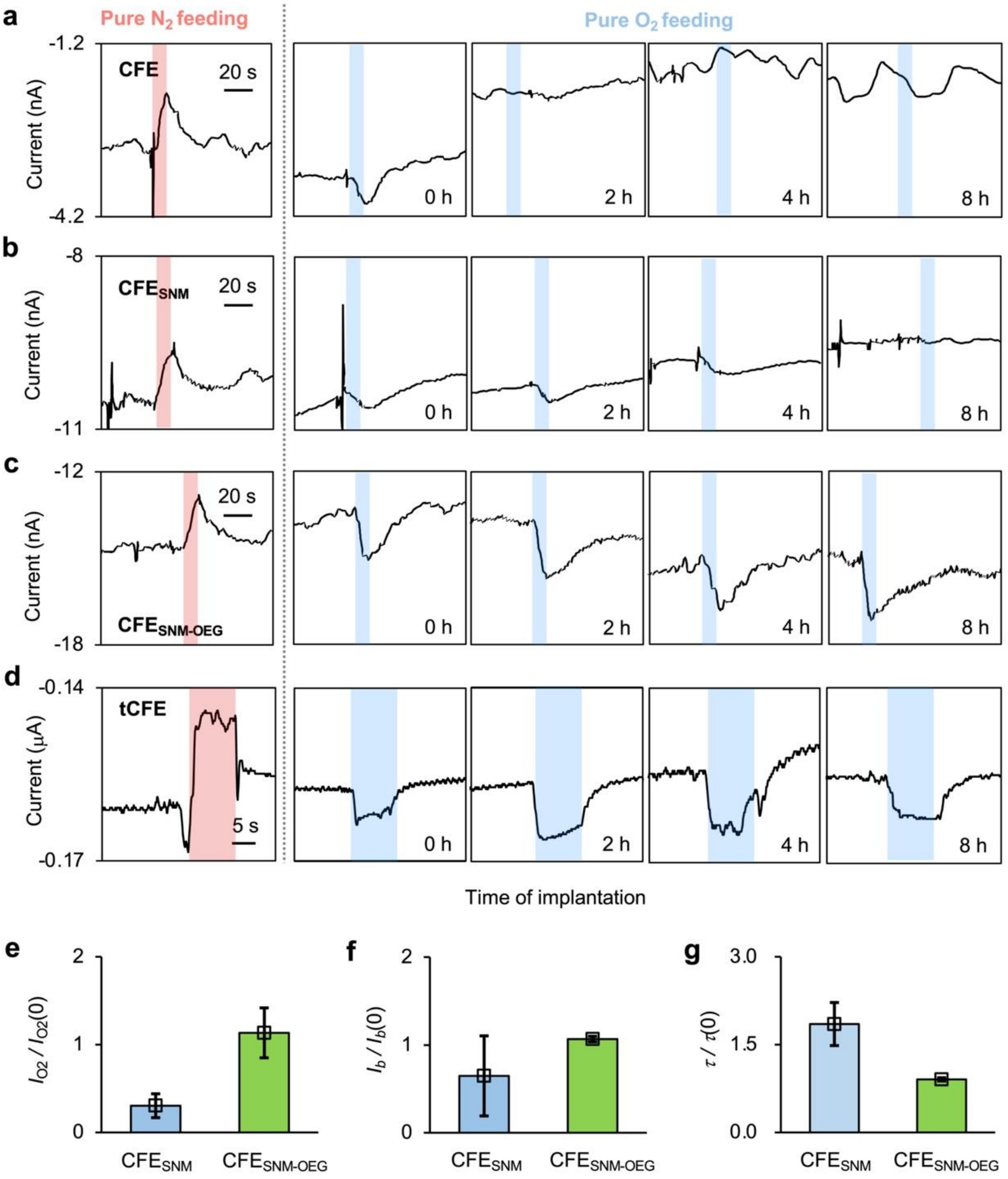

Figure S10. (**a**–**d**) Chronoamperometric curves recorded with CFE (**a**), CFE_{SNM} (**b**), $CFE_{SNM\text{-}OEG}$ (**c**) and tCFE (**d**) implanted in the hippocampus of mouse brain in response to pure nitrogen feeding (left) and intermittent pure oxygen feeding for 8 h (right). The latter was performed by giving pure oxygen atmosphere around the nose of mice for 10 s every hour. The electrode potential was biased at −0.4 V for tCFE and −0.8 V for others. (**e**–**g**) The relative variations of response current ($I_{O2}/I_{O2}(0)$, **e**), base current ($I_b/I_b(0)$, **f**) and response time ($\tau/\tau(0)$, **g**) of CFE_{SNM} and $CFE_{SNM\text{-}OEG}$ at 8 h after implantation ($n = 3$ electrodes). $I_{O2}(0)$, $I_b(0)$ and $\tau(0)$ refer to the values measured right after implantation. Data are expressed as mean ± s.d.

Pre- and post-calibration curves. The stability of microelectrodes can be evaluated by comparing calibration curves before and after implantation, often called pre-calibration and post-calibration curves, which were measured in aCSF upon the successive increase of oxygen concentration (**Figure 1k** in the manuscript and **Figure S11**). The slope of calibration curve represents the sensitivity of electrode. The ratio between the slope of post-calibration curve (S_{post}) and that of pre-calibration curve (S_{pre}), namely S_{post}/S_{pre}, was estimated to evaluate the stability and anti-biofouling ability of microelectrodes. **Figure S11a–b** compares post-calibration and pre-calibration curves obtained with CFE_{SNM} and $CFE_{SNM\text{-}OEG}$. Together with those of CFE and tCFE shown in **Figure 1k**, a sharp decrease of analytical sensitivity was observed for CFE and CFE_{SNM}. In contrast, thanks to excellent anti-biofouling ability, post-calibration curves obtained with $CFE_{SNM\text{-}OEG}$ and tCFE were close to their pre-calibration curves, keeping 78.4 ± 3.5% and 85.8 ± 3.6% of analytical sensitivity after being implanted for 8 h (**Figure S11c**).

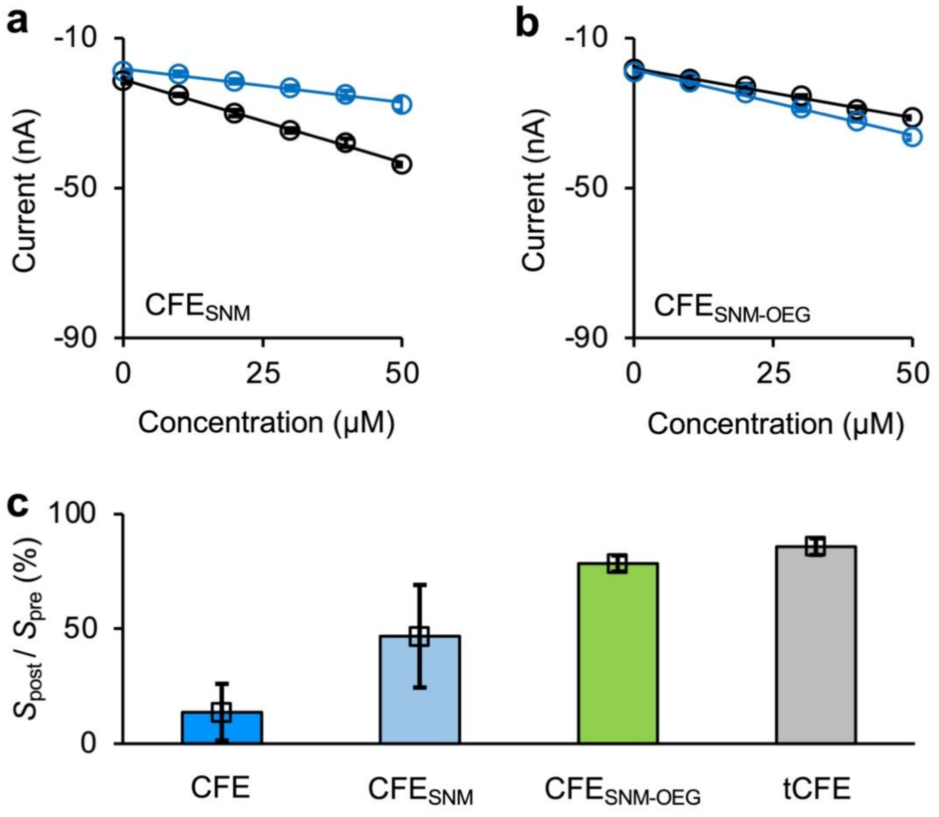

Figure S11. (**a**–**b**) Pre-calibration and post-calibration curves obtained with CFE$_{SNM}$ (**a**) and CFE$_{SNM-OEG}$ (**b**) upon the successive increase of oxygen concentration in aCSF (pH = 7.2–7.4) before implantation (black) and after being implanted in the mouse brain for 8 h (blue). (**c**) The ratio of sensitivities, S_{post}/S_{pre}, between post-calibration and pre-calibration of CFE, CFE$_{SNM}$, CFE$_{SNM-OEG}$ and tCFE. Data are expressed as mean ± s.d.

S5. Histology and immunohistochemistry

The biocompatibility of electrodes was studied by histology and immunohistochemistry.

Preparation of brain sections. Mice were anaesthetized with pentobarbital sodium (0.1 mg g^{-1}), then the microelectrode was implanted into the cortex through the stereotaxic surgery and fixed to the skull with dental cement. As a control experiment, a severe injury was mechanically made by the tip of a syringe (290 μm in diameter) to the same cortex location. After 21 days, mice with either implanted microelectrode or cerebral injury were anaesthetized and decapitated to isolate the brain. The isolated brain was sequentially fixed by 4% (vol/vol) paraformaldehyde in 0.1 M PB for 24 h, dehydrated in 20% and 30% sucrose solution at 4 °C for respective 24 h and embedded in the frozen medium. Sections with a thickness of 25 mm were cut using freezing microtome (NX50, Thermo).

Immunohistochemistry. Immunohistochemistry was conducted following a classical procedure. Firstly, frozen sections were twice rinsed with 0.025% Triton X-100 (v/v) in TBS for 5 min, followed by antigen recovery in the quick antigen retrieval solution for 5 min and rinsed again with 0.025% Triton X-100 for 3 times. They were then blocked with 10% (wt/v) goat serum and 1% (wt/v) BSA in TBS for 2 h. Subsequently, the sections were successively incubated with primary antibodies (CD11b, 1:200) for 12 h and secondary antibodies (Cy3 conjugated goat anti-rabbit IgG (H+L), 1:300) for 1 h at 4 °C, in between two incubation steps unbounded antibodies were removed by rinsing with 0.025% Triton X-100 in TBS for 5 min. Finally, sections were mounted with the commercial antifade medium containing DAPI. The confocal fluorescence images were captured on a high-resolution microscope (IX83-FV3000-OSR, Olympus).

The cerebral injury will result in the expression of CD11b proteins by inflammatory cells, which can give red fluorescence (from Cy3) after immunohistochemistry staining.[S10] However, as shown in **Figure S12a** and **Figure 1o** in the manuscript, no red fluorescence was observed around implanted CFE or tCFE, similar to that observed for the control group of normal sections (**Figure 1n** in the manuscript), indicating no immunological reaction was induced by the electrode implantation. As reported previously, carbon fiber with a small

diameter is biocompatible and does not evoke severe brain injury.[S11] In contrast, severe immunological reaction was induced by mechanical injury and overexpression of CD11b proteins was detected (**Figure S12b**). Shown in the **Figure S12c–e** is the statistical analysis of three parameters, namely the number of cells, percentage of $CD11^{+}b$ cells and average fluorescence intensity of CD11b, derived from immunohistochemical staining experiments. For sections implanted with CFE or tCFE, all three parameters are almost the same with the control group of normal sections. In the case of sections with mechanical injury, all three parameters become larger than the control group.

Optical imaging. Sections were treated with Hematoxylin and Eosin (H&E), Nissl and Masson’s Trichrome staining solutions, respectively, subsequently mounted with the neutral balsam and observed with an inverted optical microscope (cx21, Nikon). As shown in **Figure 1p–u** in the manuscript and **Figure S12f–k**, the sections with implanted microelectrodes are similar to the normal ones (**Figure 1p–u** in the manuscript and **Figure S12f–h**), whereas an obvious decrease of the number of neural cells and formation of scar are observed on the section with mechanical injury (**Figure S12i–k**).

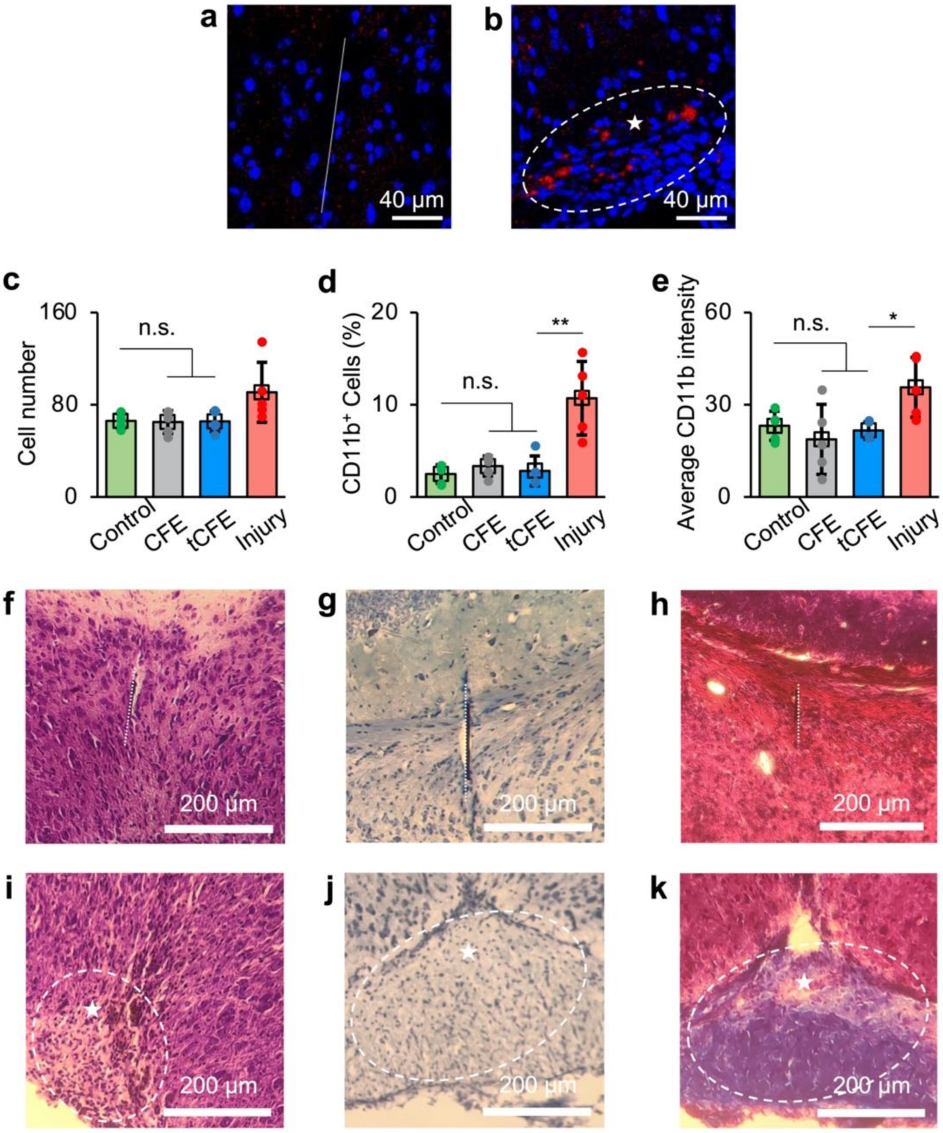

Figure S12. (**a**–**b**) Confocal fluorescence images of immunohistochemical staining of CD11b proteins on sections with CFE implantation (**a**) and mechanical injury (**b**). CD11b proteins are seen in red (from Cy3) and the nucleus in blue (from DAPI). The white line and dashed ellipse annotate the locations of implanted CFE and mechanical injury, respectively. **c-e** Statistical analysis of the number of cells, the percentage of $CD11^{+}b$ cells and the average fluorescence intensity of CD11b for normal sections (control), sections implanted with CFE and tCFE and those with mechanical injury (n = 5 mice). Data are expressed as mean ± s.d. Significance was determined by two-tailed unpaired Student's *t*-test (*$p < 0.05$, **$p < 0.01$ and n.s., no significance). **f**–**k** Optical images of H&E (**f**, **i**), Nissl (**g**, **j**) and Masson's trichrome staining (**h**, **k**) of sections with CFE implantation (**f**–**h**) and mechanical injury (**i**–**k**). The white dotted line indicates the location of implanted tCFE. The white dashed circle with star indicates the position of mechanical injury. Each group was repeated for more than five times.

S6. The EPE system

Figure S13 shows the EPE system, including a digital stereotaxic platform for surgery, an electrochemical workstation and three electrodes for electroanalysis, a cervical oximeter for recording physiological parameters (such as the heart rate, breath rate and arterial oxygen saturation) and a video camera for convulsion observation. In addition, ethological tests were performed to investigate the influence of caffeine on mice behaviors. The hardware used in different ethological tests are shown at the bottom.

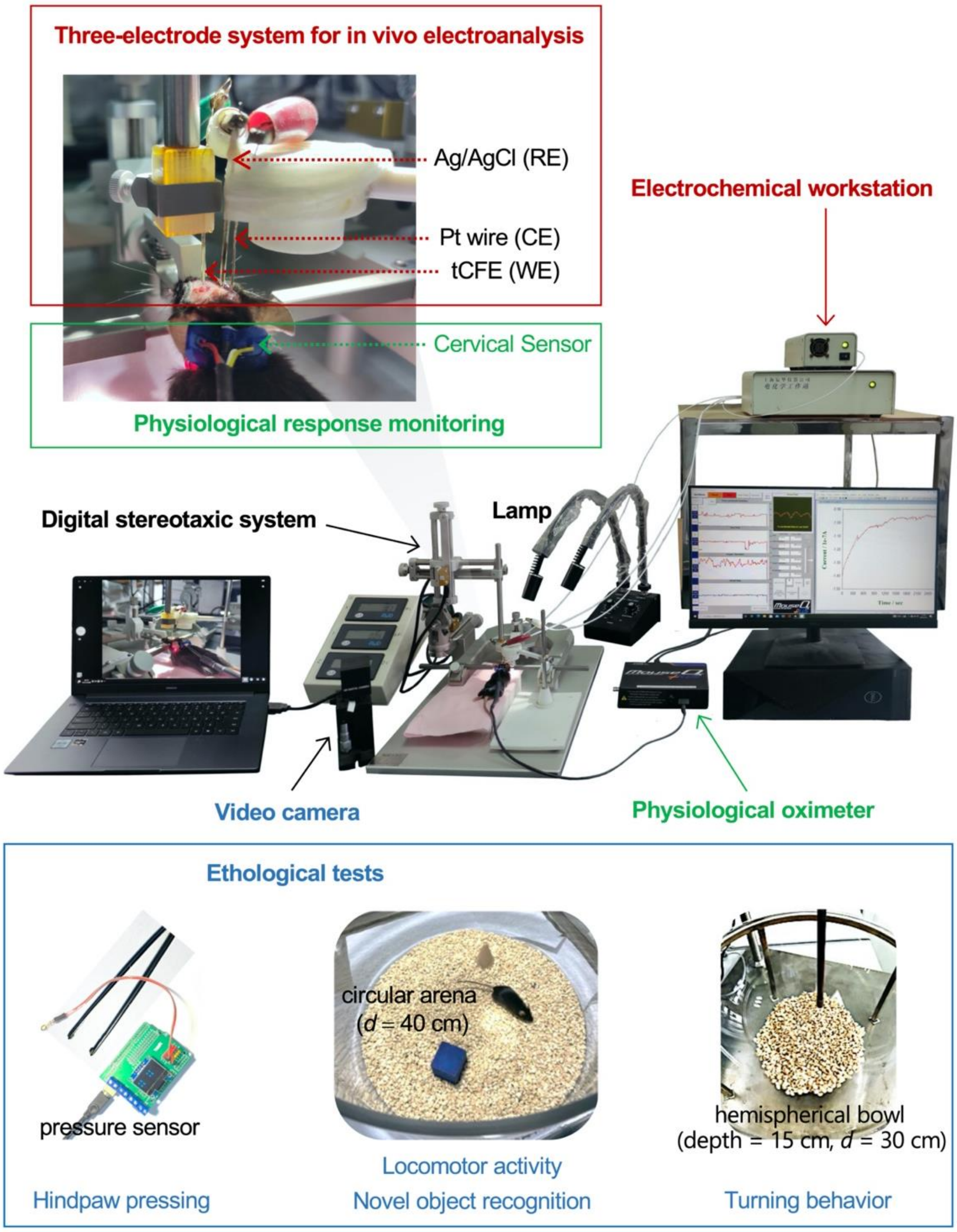

Figure S13. Photograph of the EPE experimental system.

S7. Typical physiological responses

Figure S14 shows typical physiological responses (including the heart rate, breath rate and arterial oxygen saturation) of mice under high-dose caffeine, low-dose caffeine and saline stimulation. **Figures S15–18** display the relative heart rate variation, namely $(HR-HR_b)/HR_b$ (the variation of heart rate relative to the base heart rate at the neutral state level), the relative breath rate variation, namely $(BR-BR_b)/BR_b$ (the variation of breath rate relative to the base breath rate at the neutral state level) and relative arterial oxygen saturation variation, namely $(AOS-AOS_b)/AOS_b$ (the variation of arterial oxygen saturation relative to the base arterial oxygen saturation at the neutral state level) measured at specific times/stages while recording the current responses in different brain regions.

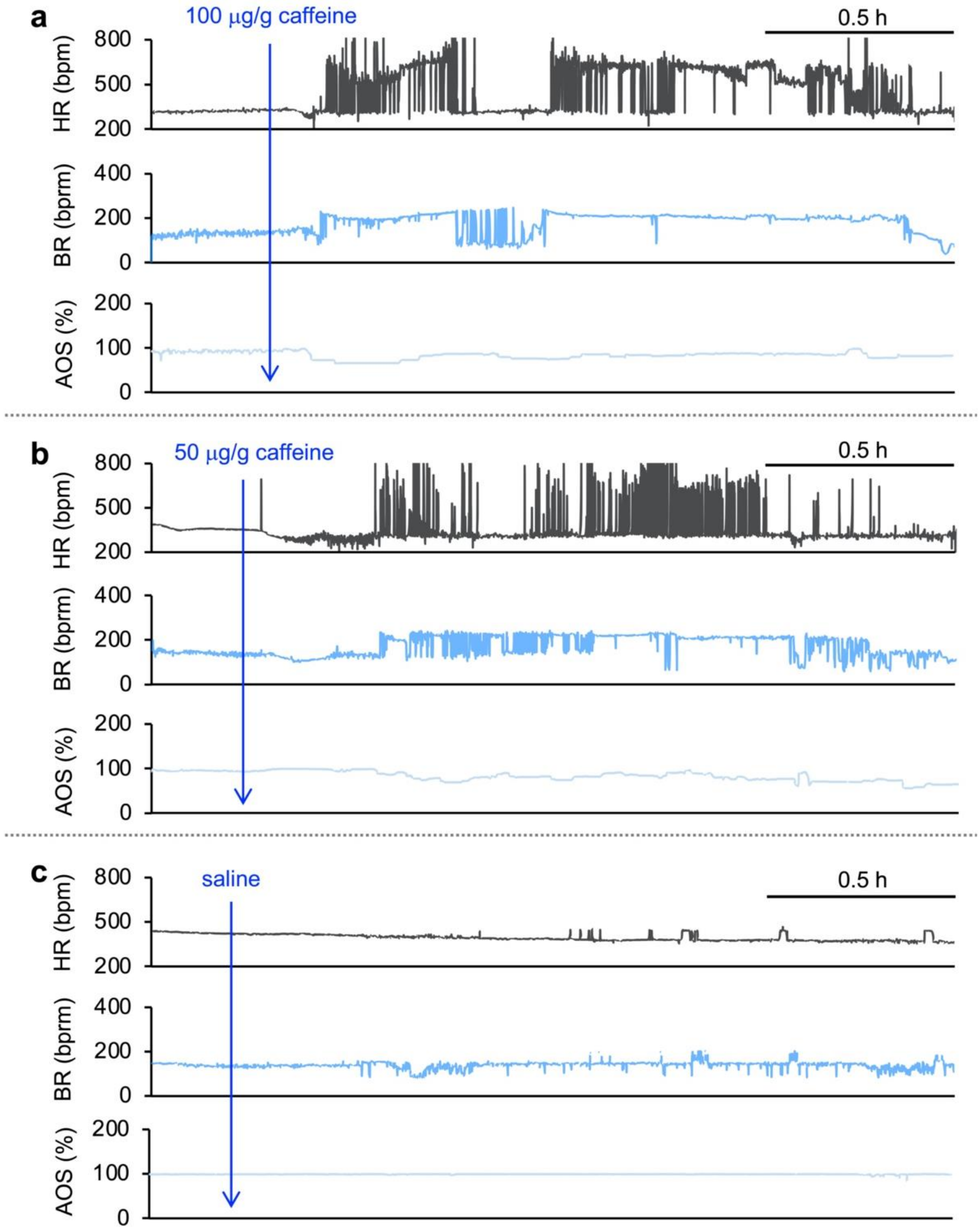

Figure S14. The variations of heart rate (HR), breath rate (BR) and arterial oxygen saturation (AOS) recorded with the cervical sensor under high-dose caffeine (**a**), low-dose of caffeine (**b**) and saline (**c**) stimulation.

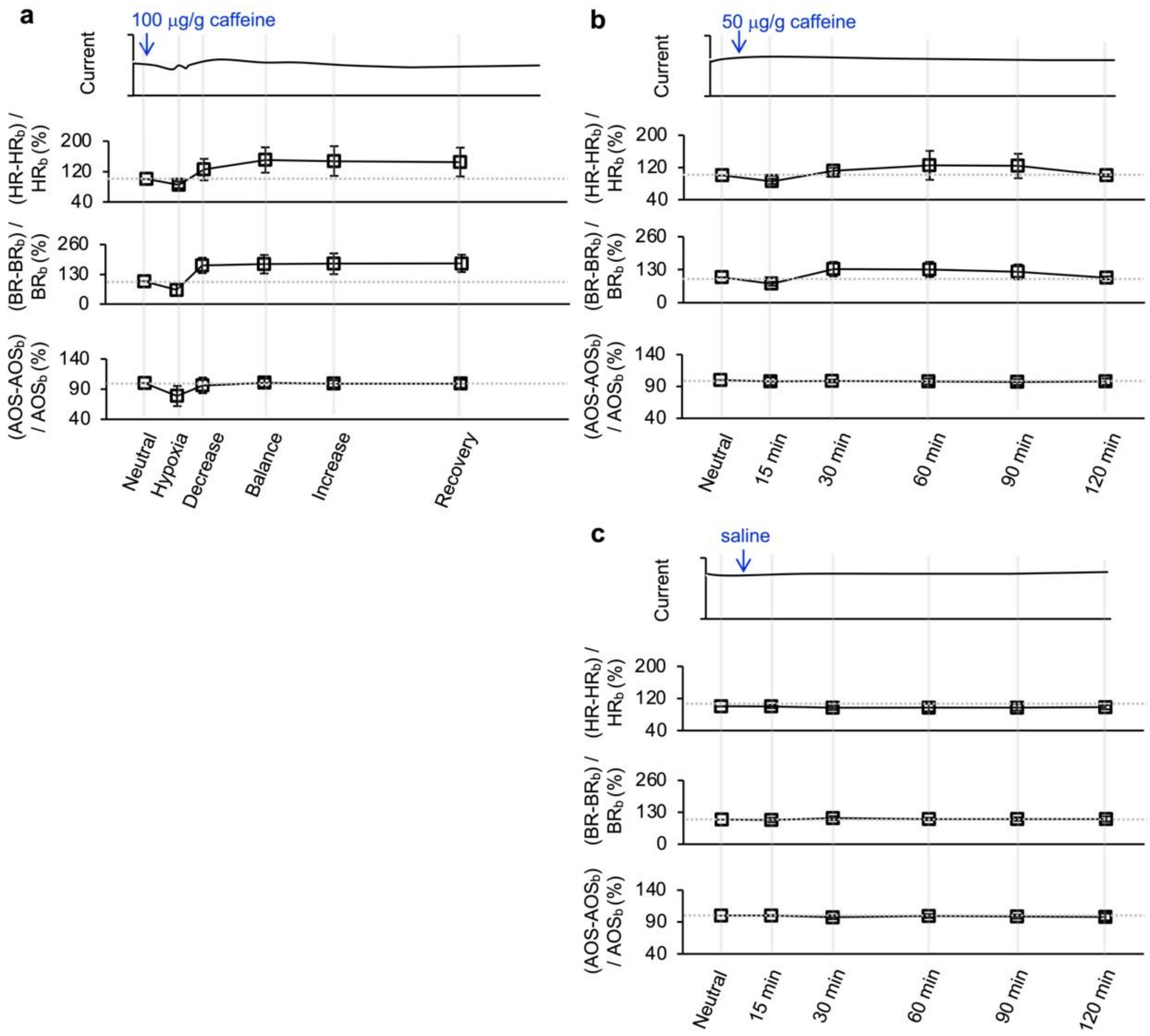

Figure S15. Relative variations of three physiological parameters at specific stages/times of chronoamperometric current curves measured in S1 under high-dose caffeine (**a**), low-dose caffeine (**b**) and saline (**c**) stimulation ($n = 8$ mice). Data are expressed as mean ± s.d.

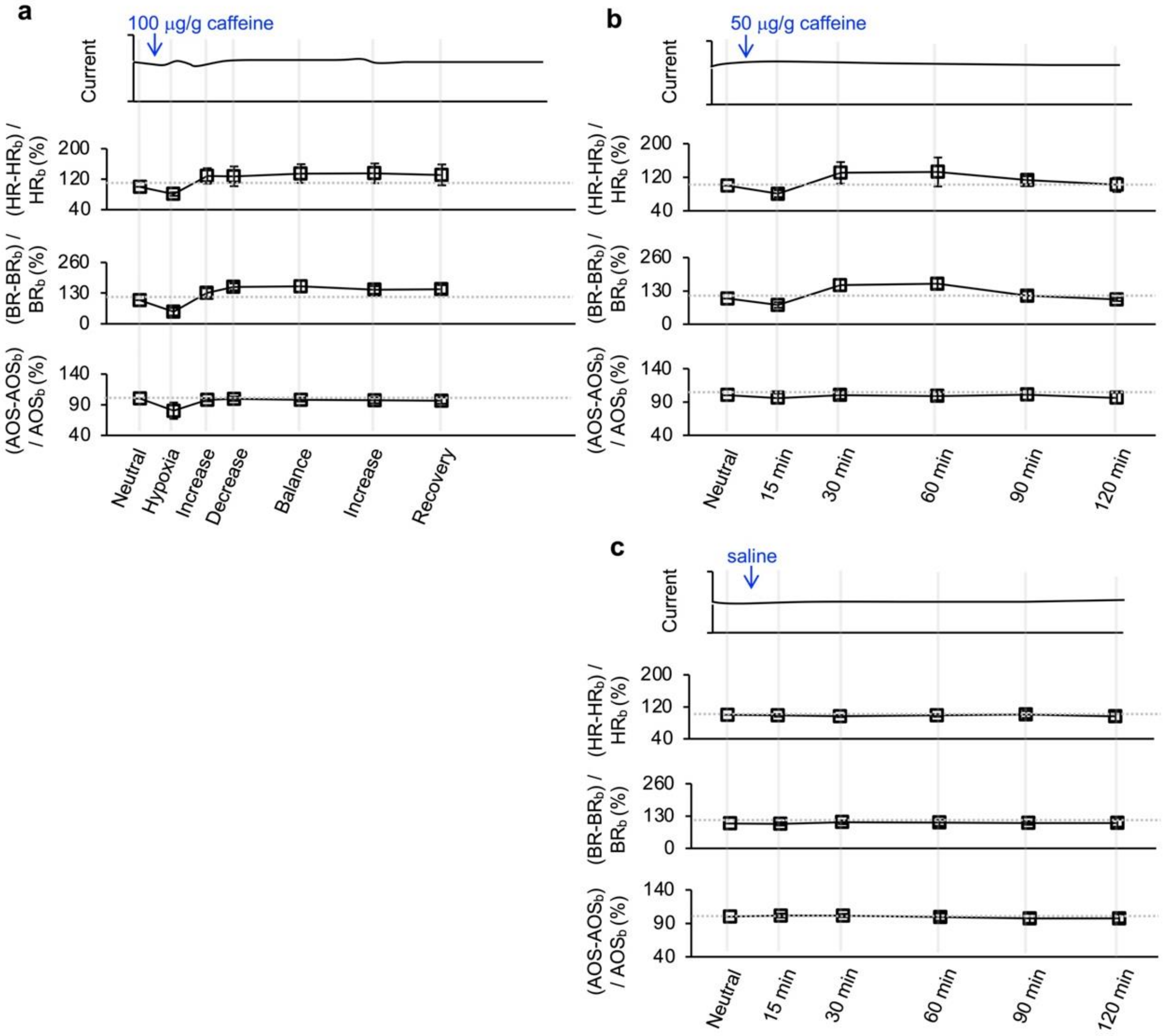

Figure S16. Relative variations of three physiological parameters at specific stages/times of chronoamperometric current curves measured in M1 under high-dose caffeine (**a**), low-dose caffeine (**b**) and saline (**c**) stimulation (n = 8 mice). Data are expressed as mean ± s.d.

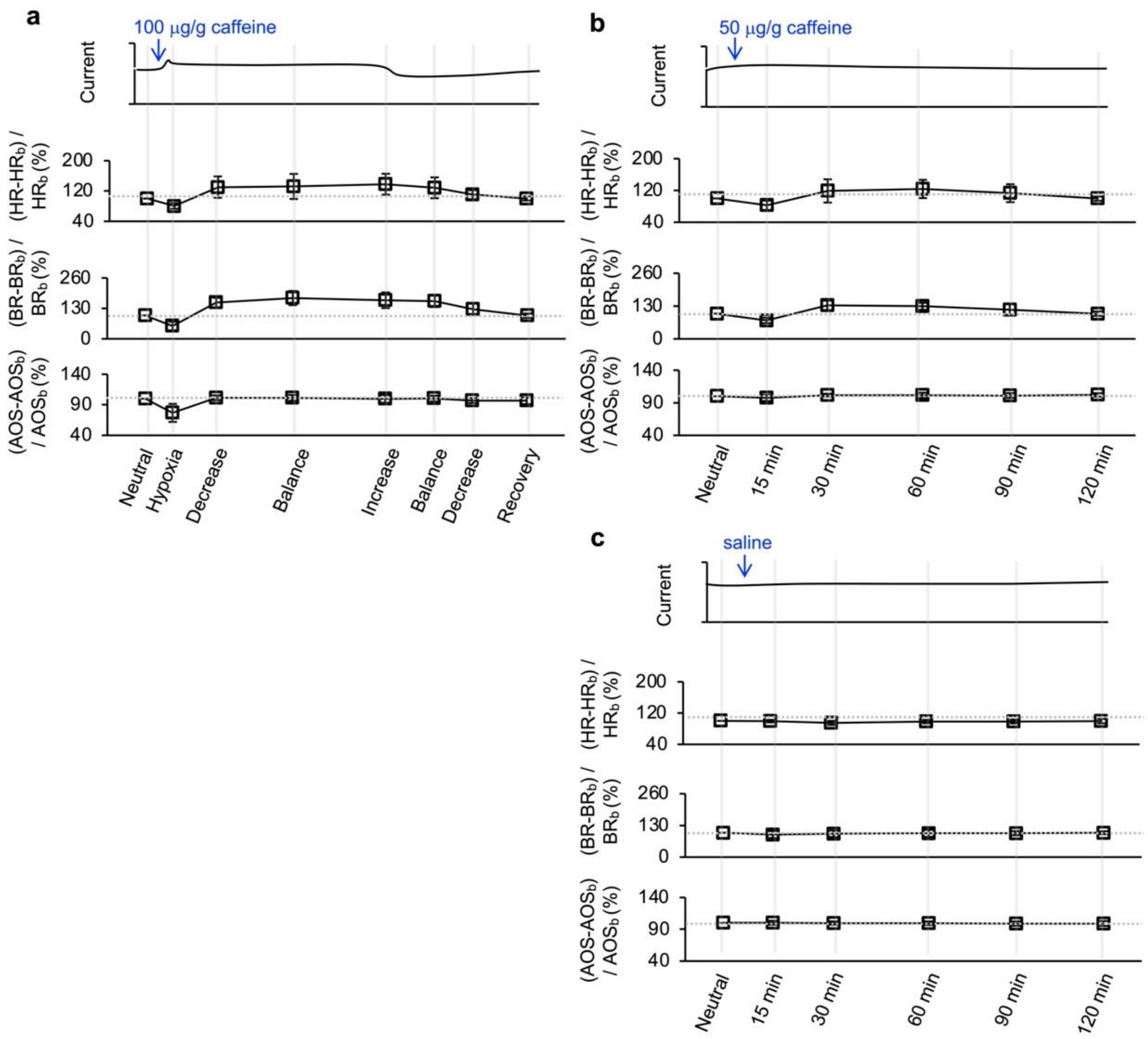

Figure S17. Relative variations of three physiological parameters at specific stages/times of chronoamperometric current curves measured in the hippocampus under high-dose caffeine (**a**), low-dose caffeine (**b**) and saline (**c**) stimulation (n = 8 mice). Data are expressed as mean ± s.d.

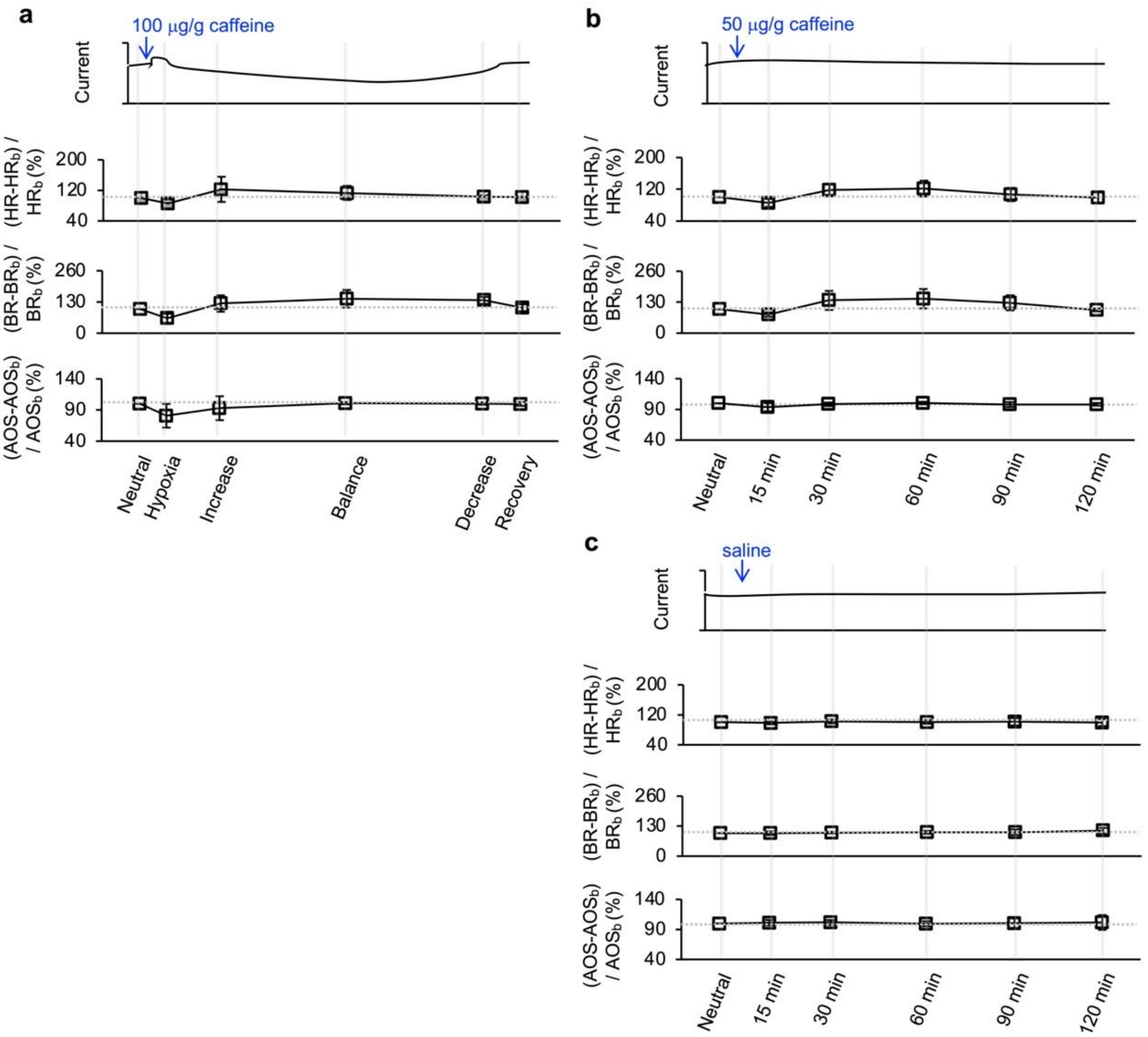

Figure S18. Relative variations of three physiological parameters at specific stages/times of chronoamperometric current curves measured in the striatum under high-dose caffeine (**a**), low-dose caffeine (**b**) and saline (**c**) stimulation (n = 8 mice). Data are expressed as mean ± s.d.

S8. Long-term current variation in the hippocampus

In the chronoamperometric curve recorded in the hippocampus of mouse brain (top-right of **Figure 2a**), we can clearly see that after the hypoxia the magnitude of oxygen reduction current firstly increased and then decreased over time. The current variations in these two periods are termed as stage I and stage II. The average duration of two stages takes up ~59 ± 17% and ~41 ± 18%, respectively, of the total t_{O2} (**Figure S19**).

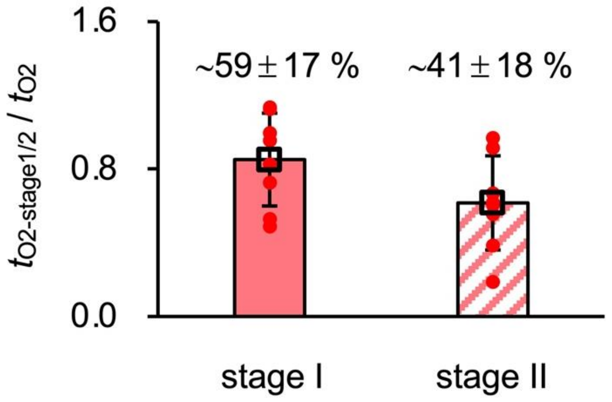

Figure S19. The percentage of duration of current variation at stage I and stage II in the total t_{O2}. The duration values were determined from chronoamperometric curves recorded in the hippocampus under high-dose caffeine stimulation (n = 8 mice). Data are expressed as mean ± s.d.

S9. Responses under low-dose caffeine stimulation

Figure S20 show chronoamperometric curves measured in four brain regions under low-dose caffeine and saline stimulation. The current is featureless in comparison with that shown in **Figure 2a**.

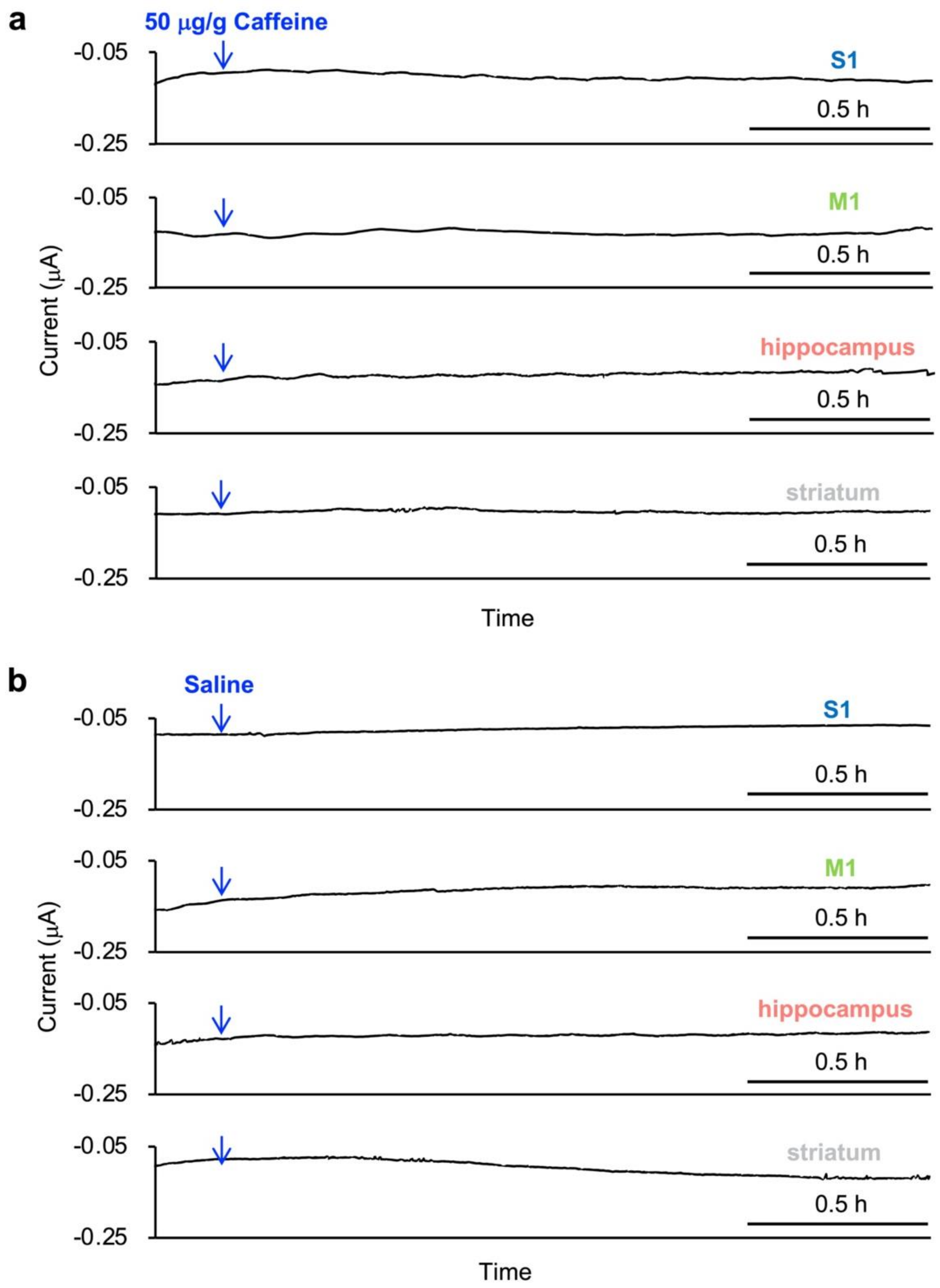

Figure S20. Typical chronoamperometric responses recorded in S1, M1, hippocampus and striatum under low-dose caffeine (**a**) and saline (**b**) stimulation.

Figure S21 displays $(I_{O_2}-I_b)/I_b$, t_{O_2} and t_{phy} in four brain regions under low-dose caffeine and saline stimulation. No remarkable change of former two parameters was observed, although t_{phy} varied a lot under low-dose caffeine stimulation.

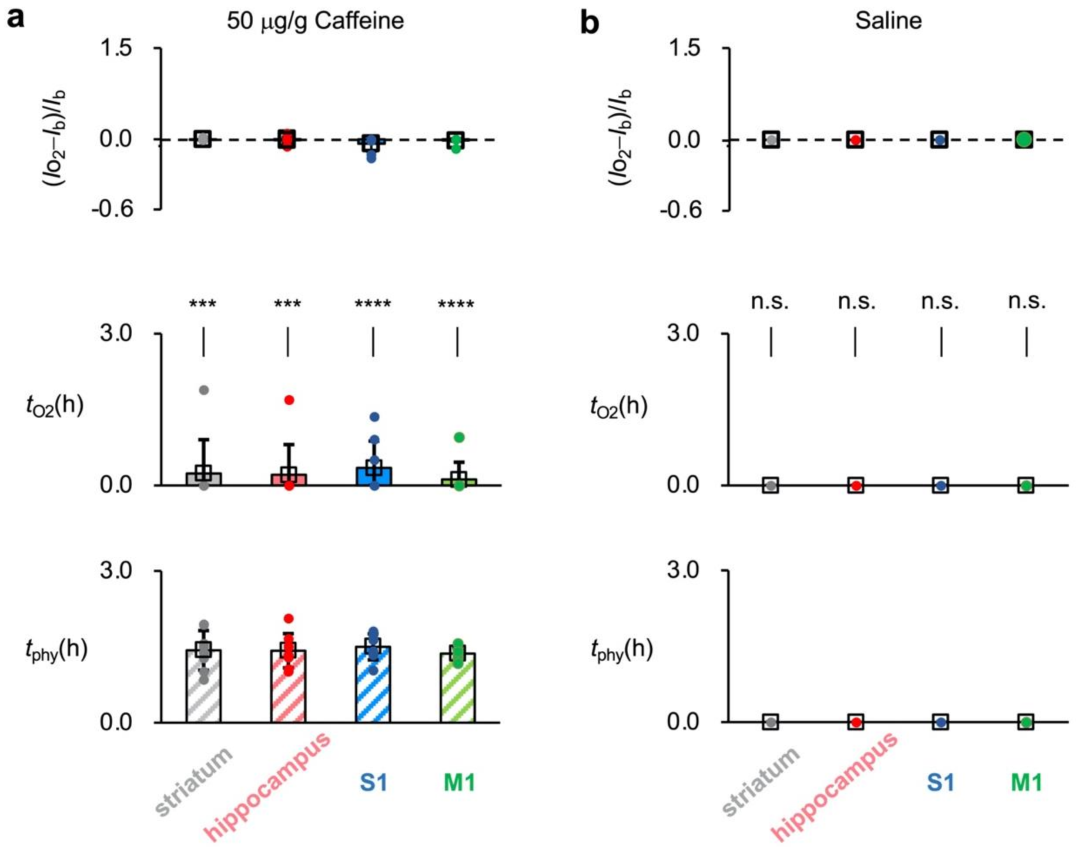

Figure S21. $(I_{O_2}-I_b)/I_b$, t_{O_2} and t_{phy} in four brain regions under low-dose caffeine (**a**) and saline stimulation (**b**) (n = 8 mice). Data are expressed as mean ± s.d. Significance was determined by two-tailed unpaired Student's t-test (***$p < 0.001$, ****$p < 0.0001$ and n.s., no significance).

S10. Caffeine induced brain hypoxia

Figure S22a–c shows chronoamperometric and physiological responses recorded in M1, hippocampus and striatum of the brain of living mice during the acute hypoxia induced by high-dose caffeine injection. The corresponding response time is summarized in **Figure S22d–f.** Similar to that observed in S1 (**Figure 2c–d**), the concurrent decrease of current and arterial oxygen saturation, as well as the inhibition of heart and breath rate, was also detected in three brain regions. In contrast, although the hypoxia was not induced by low-dose caffeine injection, the inhibition of heart rate and breath rate was still clear (**Figure S23**). A control experiment showed that the saline did not evoke the hypoxia (**Figure S24**).

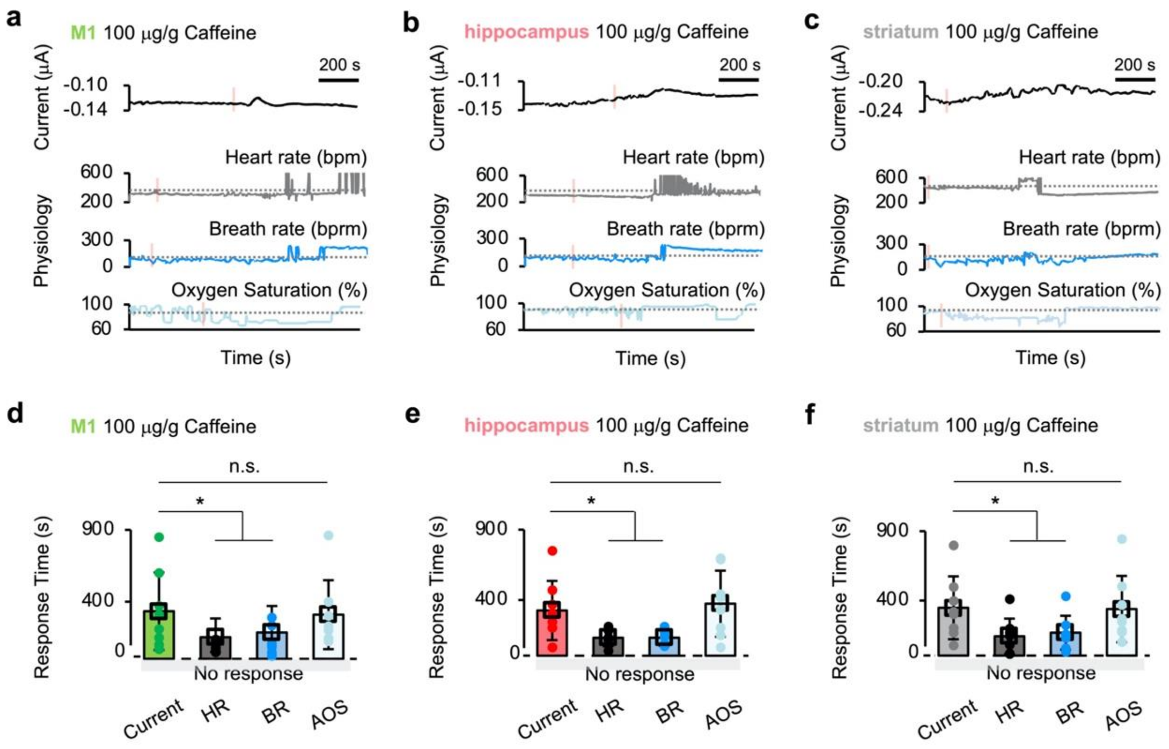

Figure S22. (**a–c**) Typical chronoamperometric curves recorded in M1 (**a**), hippocampus (**b**) and striatum (**c**) and concurrent physiological responses during hypoxia induced by high-dose caffeine injection. The pink lines indicate the moments at which the variations were detected. (**d–f**) Statistical analysis of the corresponding response time of current and heart rate (HR), breath rate (BR) and arterial oxygen saturation (AOS) (n = 8 mice). Data are expressed as mean ± s.d. Significance was determined by two-tailed unpaired Student's t-test (*$p < 0.05$ and n.s., no significance).

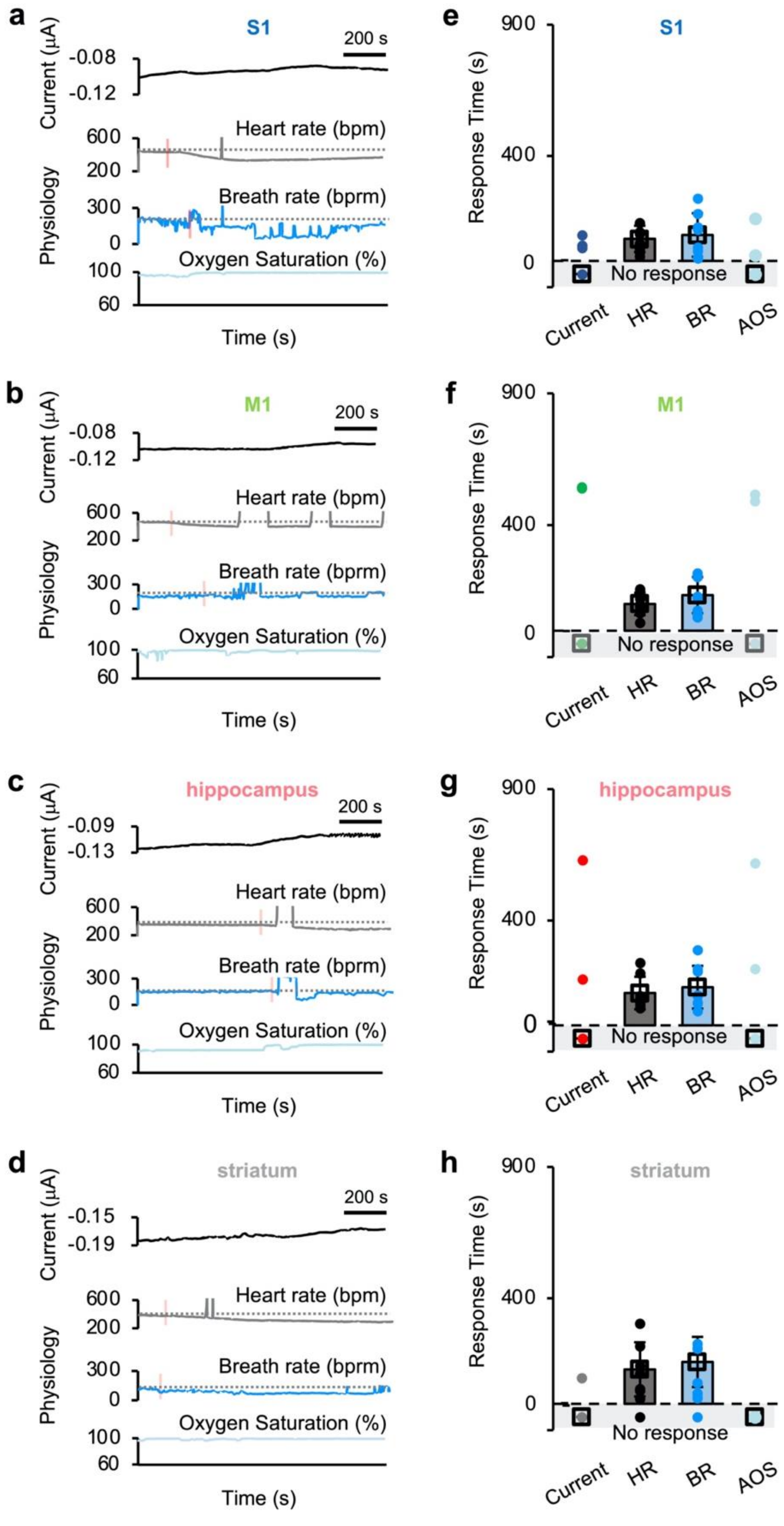

Figure S23. (**a**−**d**) Typical chronoamperometric curves recorded in four brain regions and concurrent physiological responses under low-dose caffeine stimulation. The pink lines indicate the moments at which the variations were detected. (**e**–**h**) Statistical analysis of the corresponding response time of current and physiological parameters (*n* = 8 mice). Data are expressed as mean ± s.d.

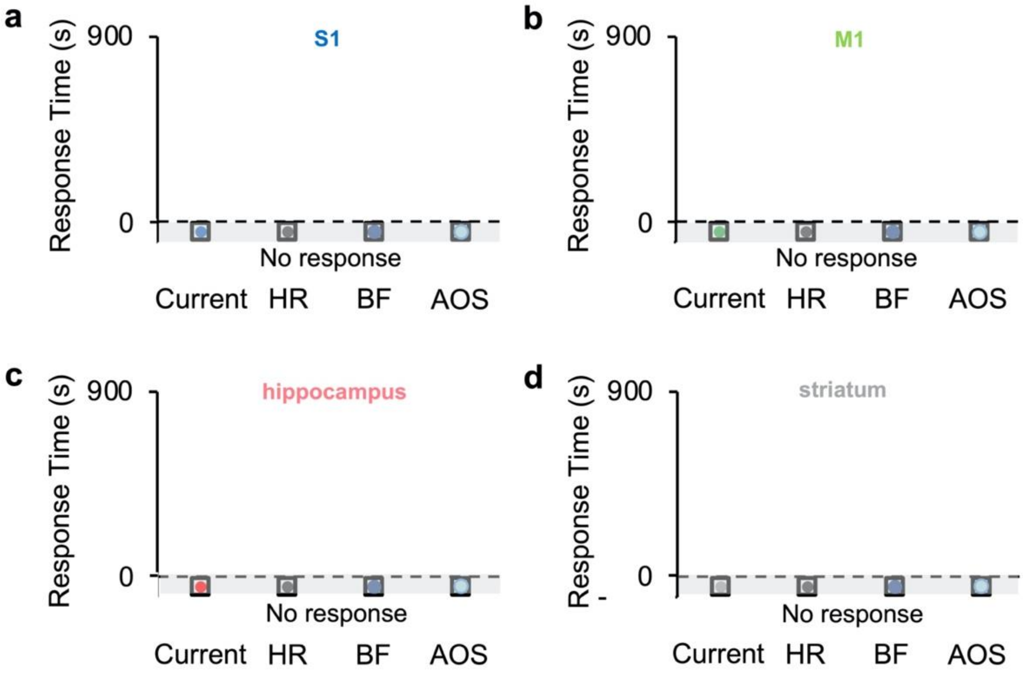

Figure S24. Statistical analysis of the response time of current measured in four brain regions under saline stimulation and that of concurrent physiological responses (n = 8 mice). Data are expressed as mean ± s.d.

S11. Hindpaw mechanical stimulation test

Figure S25a–b displays the procedure and photograph of hindpaw stimulation test. The right panel of **Figure S25b** shows the pressure-time curve. **Figure S25c–d** compares the paw withdrawal latency (PWL) of mice in response to mechanical stimulation after low-dose caffeine and saline injection, showing that the variation of PWL was insignificant and thus indicating that the activity of S1 was not affected.

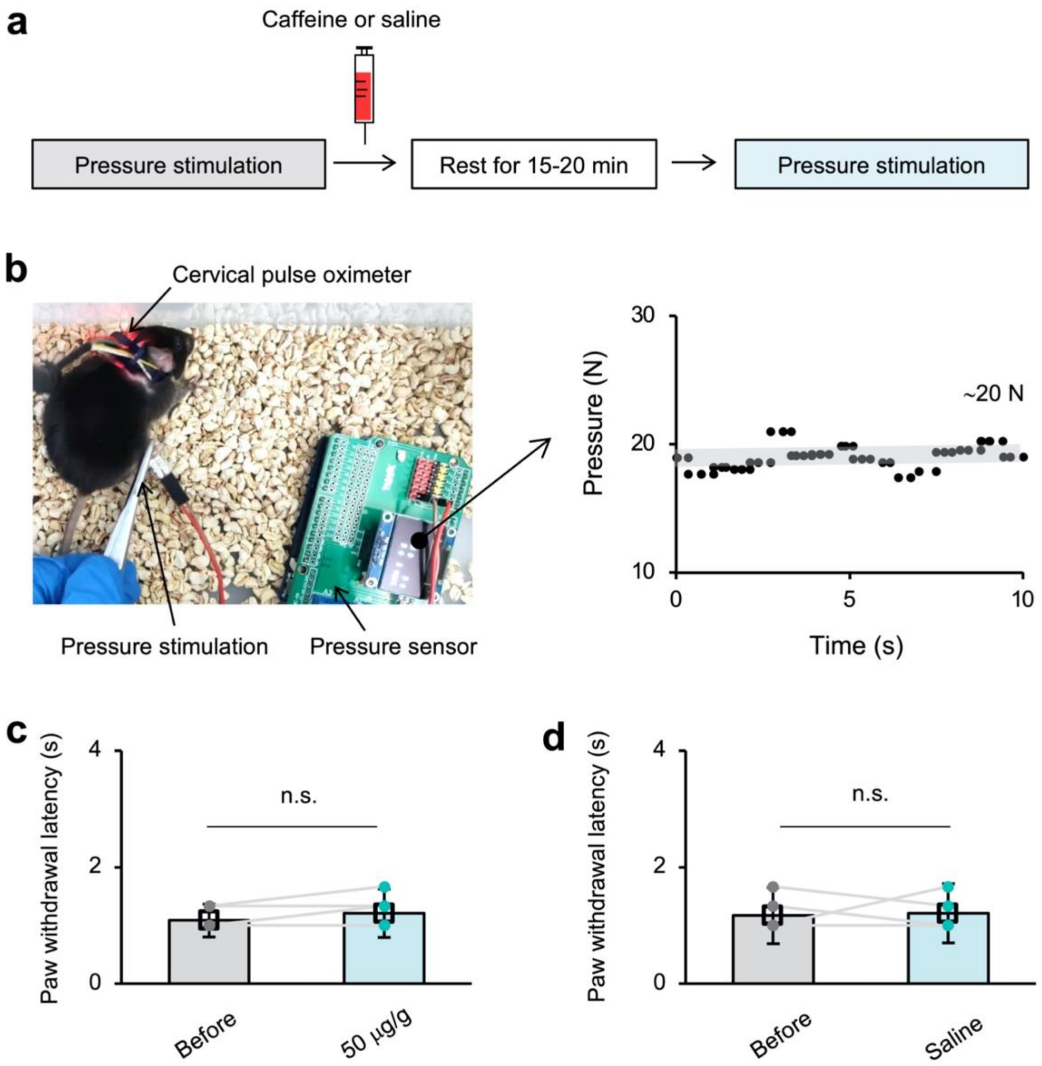

Figure S25. (**a**) The procedure of hindpaw mechanical stimulation test. (**b**) The photographic illustration of mechanical hindpaw stimulation test (left) and the pressure-time curve recorded with the pressure sensor (right). (**c–d**) The paw withdrawal latency (PWL) of mice before and after low-dose caffeine (**c**) and saline (**d**) injection (n = 8 mice). Data are expressed as mean ± s.d. Significance was determined by two-tailed unpaired Student's t-test (n.s., no significance).

S12. Locomotor activity test

The procedure of locomotor activity test is illustrated in **Figure S26a**. The total distance moved (*L*) and tracks before and after low-dose caffeine and saline injection (**Figure S26b–c**) show that the variations of *L* and motion track were insignificant, thus suggesting the activity of M1 was not affected.

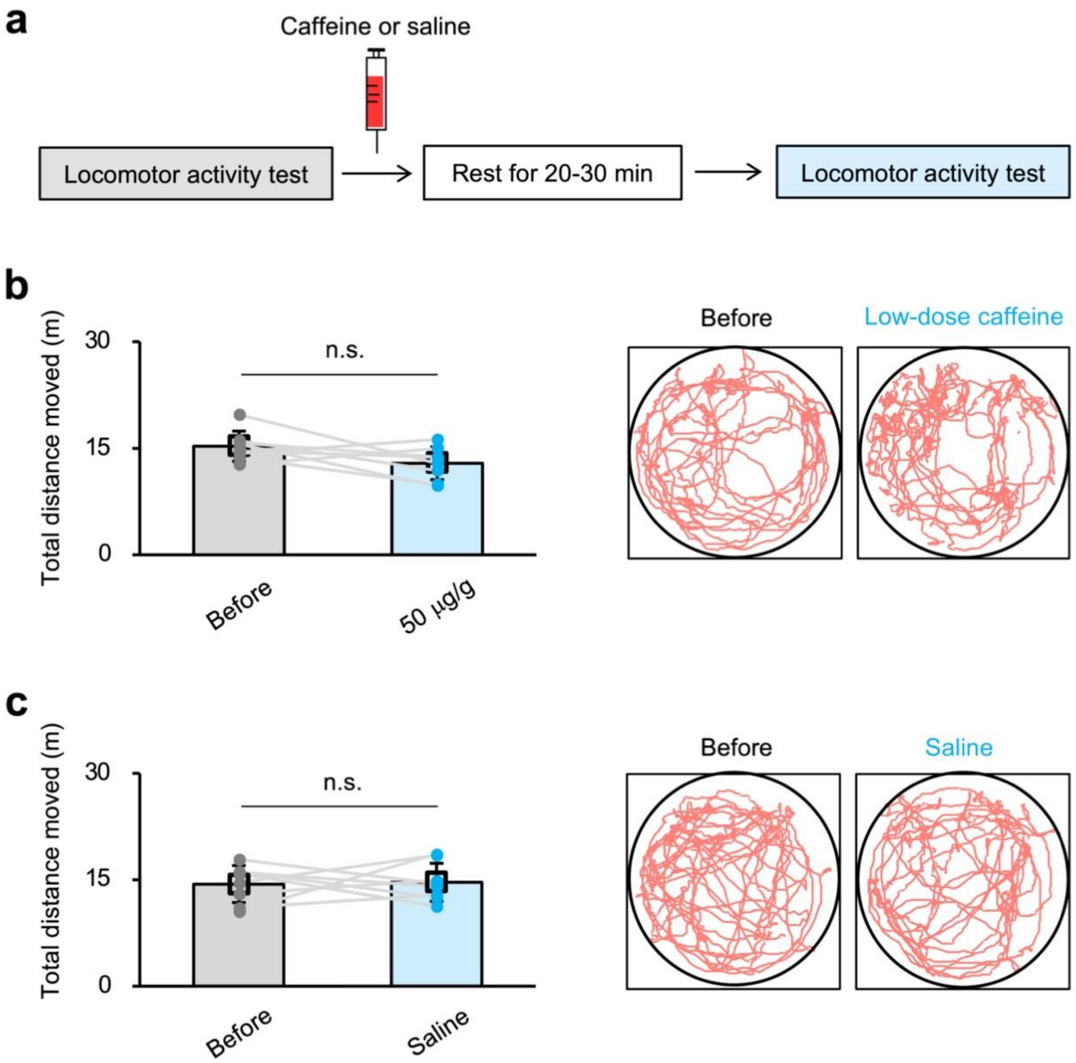

Figure S26. (**a**) The procedure of locomotor activity test. (**b–c**) The total distance that mice moved (*L*) before and after injection of low-dose caffeine (**b**) and saline (**c**) (*n* = 8 mice). The motion tracks of 5 min in a circular arena are shown on the right. Data are expressed as mean ± s.d. Significance was determined by two-tailed unpaired Student's *t*-test (n.s., no significance).

S13. New object recognition (NOR) test

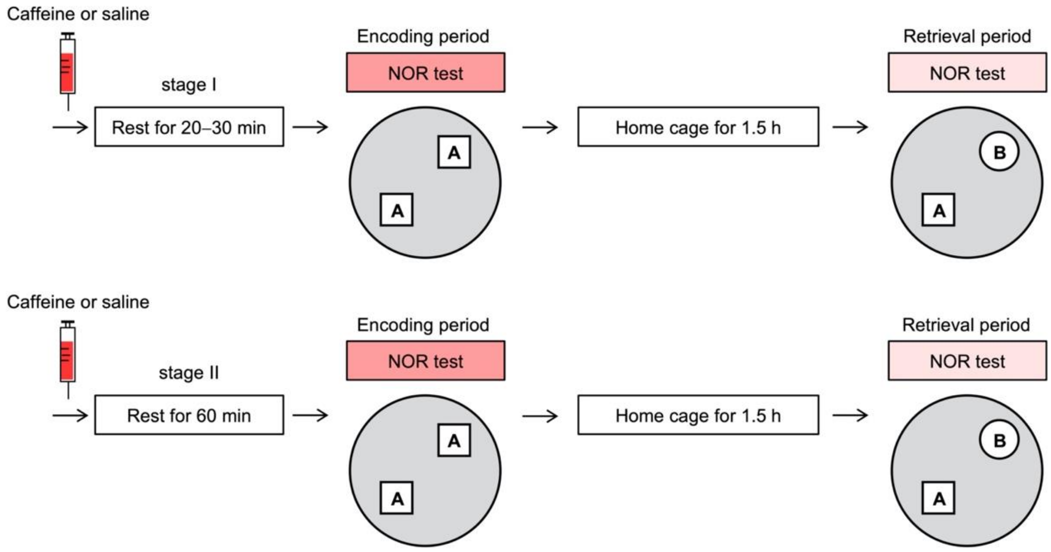

Figure S27. The procedure of new object recognition (NOR) test.

S14. Turning behavior test

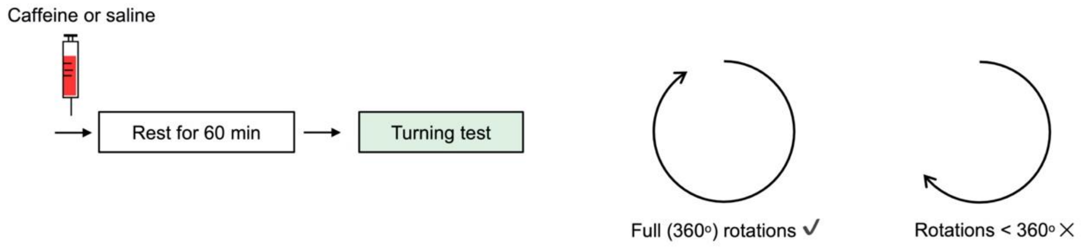

Figure S28. The procedure of turning behavior test.

S15. The convulsion

Apart from hypoxia-induced current decrease, additional current feature is also observed in the hippocampus and striatum (annotated by blue bars in **Figure 2a**), which is found to be accompanied by the convulsion of mouse (**Movie 1**). A comparison of digital images of mouse before and in the convulsion shows that neither tCFE nor the head of mouse was moved (**Figure S29c**), indicating the current feature did not originate from the noise but most likely the convulsion-induced respiratory depression. Indeed, as shown in **Figure S29a–b**, the current decrease is accompanied by a severe inhibition of breath rate and an instant decrease of arterial oxygen saturation, which are apparently different from the characteristics of brain hypoxia.

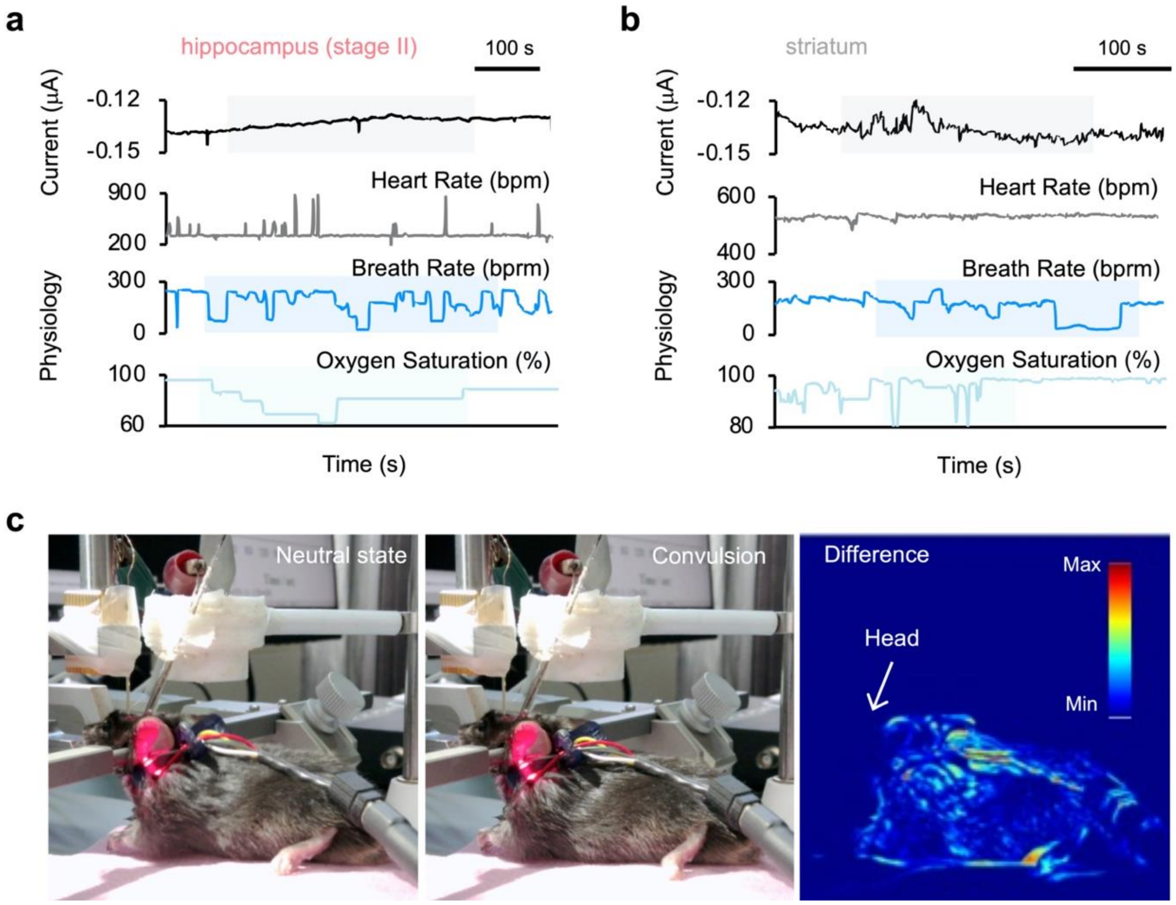

Figure S29. (**a-b**) Chronoamperometric curves and concurrent physiological responses recorded in the hippocampus (stage II) (**a**) and striatum (**b**) under high-dose caffeine stimulation. (**c**) Photographs of anesthetized mouse at the neutral state (left), in the convulsion (middle) and the difference of two images (right).

Figure S30a shows typical chronoamperometric curves and concurrent physiological responses measured in S1, M1 and hippocampus (stage I) under high-dose caffeine stimulation, showing the variations of breath rate and oxygen saturation but not the current. **Figure S30b** shows that the numbers of convulsion-induced current decrease in S1 and M1 are much less than those in the striatum and hippocampus under high-dose caffeine stimulation. Control experiments showed that low-dose of caffeine and saline did not induce convulsion.

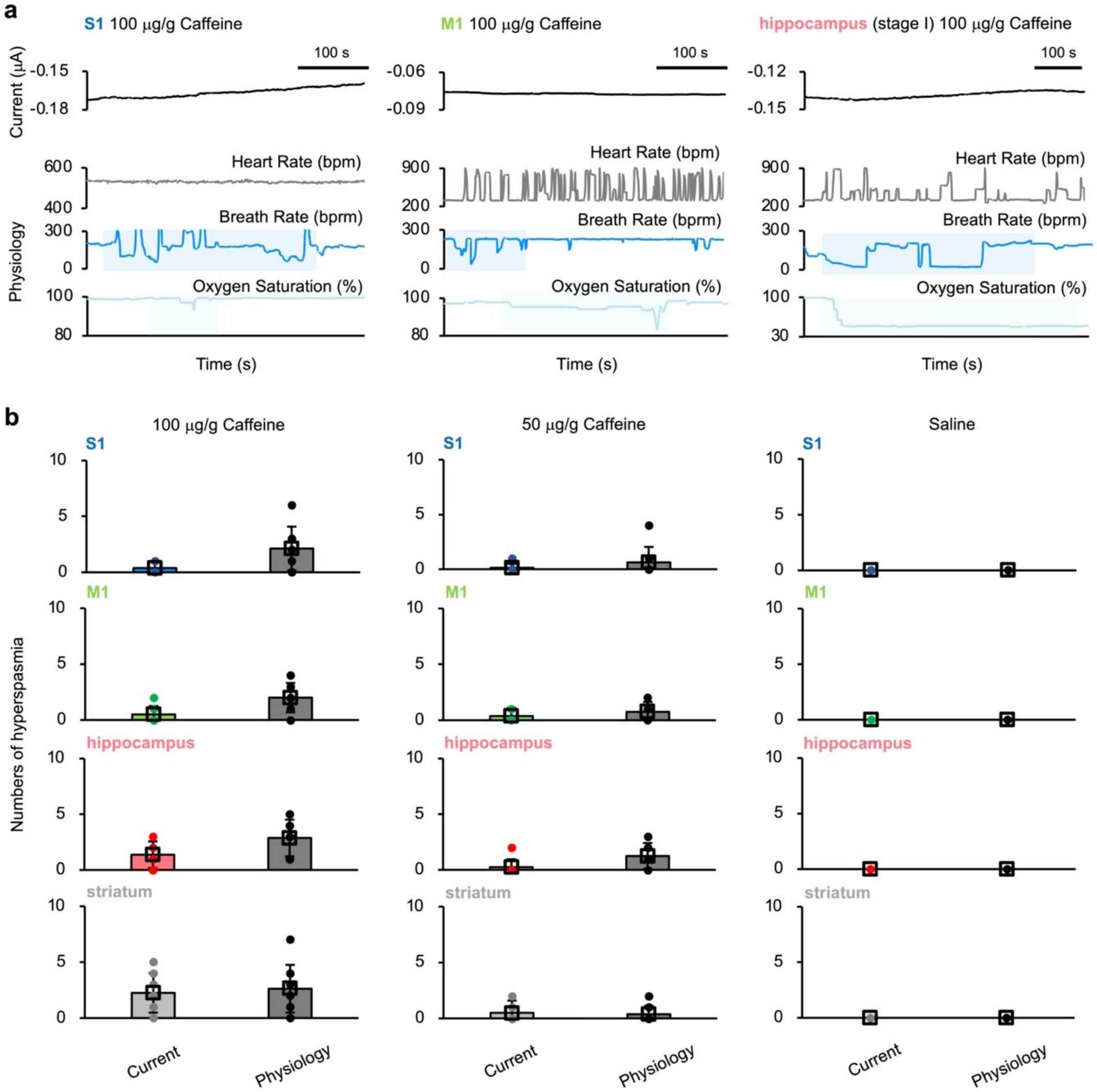

Figure S30. (**a**) Typical chronoamperometric curves and concurrent physiological responses recorded in S1, M1 and hippocampus (stage I) under high-dose caffeine stimulation. (**b**) The numbers of convulsion counted from current and physiological responses recorded in four brain regions under high-dose caffeine (left), low-dose caffeine (middle) and saline (right) stimulation (n = 8 mice). Data are expressed as mean ± s.d.

S16. Neuronal damage induced by long-term caffeine intake

Wireless electrochemical system. It consists of two hardware units, namely a custom potentiostat with potential control and data acquisition module on the flexible printed circuit board (FPCB) and a data processing and transmission module on the printed circuit board (PCB). FPCB and PCB have reserved program debugging interfaces. The personal computer with a user interface controls the working procedures of FPCB and PCB through JTAG port. **Figure S31a** demonstrates the workflow of wireless electrochemical measurement. The preprogrammed microcontroller unit (MCU) works according to the command input by computer through Bluetooth transceiver to drive the digital to analog converter (DAC). Then a voltage is output and applied on the microelectrode through operational amplifier (OPA) to perform chronoamperometry. The current signal is firstly converted into an analog voltage by the transimpedance amplifier (TIA) circuit, which is then read as a digital voltage signal by an analog to digital converter (ADC), transmitted back to MCU and wirelessly to the user interface on computer via Bluetooth. Main electronic components used in the custom potentiostat are shown in **Table S2**.

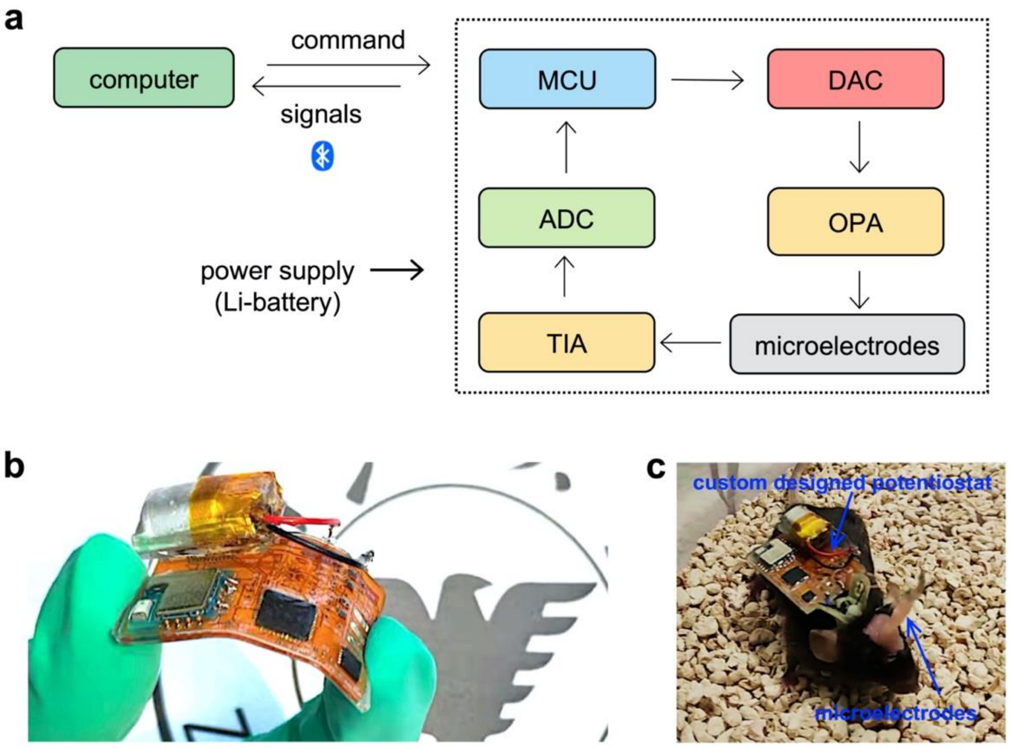

Figure S31. (**a**) The block diagram and workflow of wireless electrochemical measurement. (**b**) The custom flexible potentiostat powered by a lithium battery. (**c**) Photograph of in vivo electroanalysis on a freely-moving mouse with tCFE implanted in brain and potentiostat carried on the back of mouse.

Table S2. Main electronic components used in the custom flexible potentiostat.

Electronic components		Manufacturer
microcontroller unit (MCU)	MSP430FR5959	Texas Instrument Semiconductor Co. Ltd
digital to analog converter (DAC)	DAC8562	
analog to digital converter (ADC)	ADS1115	
Li-battery	401015	Dajia Manyi Technology Co. Ltd
Bluetooth	WH-BLE103	Youren Tech. Inc
operational amplifier (OPA)	AD8608	Analog Devices. Inc

The effect of surgery on mice. The effect of surgery on mice was investigated by monitoring the motor activity of mice. Two tCFEs were implanted in S1 and fixed onto the skull with dental cement. As shown in **Figure S32**, after surgery the motor activity of mice is remarkably inhibited with an apparent decrease of total motion distance in 2 days. Meanwhile, an increase of grooming times is also observed. But mice can entirely recover from the surgery after 4 days. Therefore, prior to the long-term caffeine stimulation study, mice were allowed to recover over 7 days.

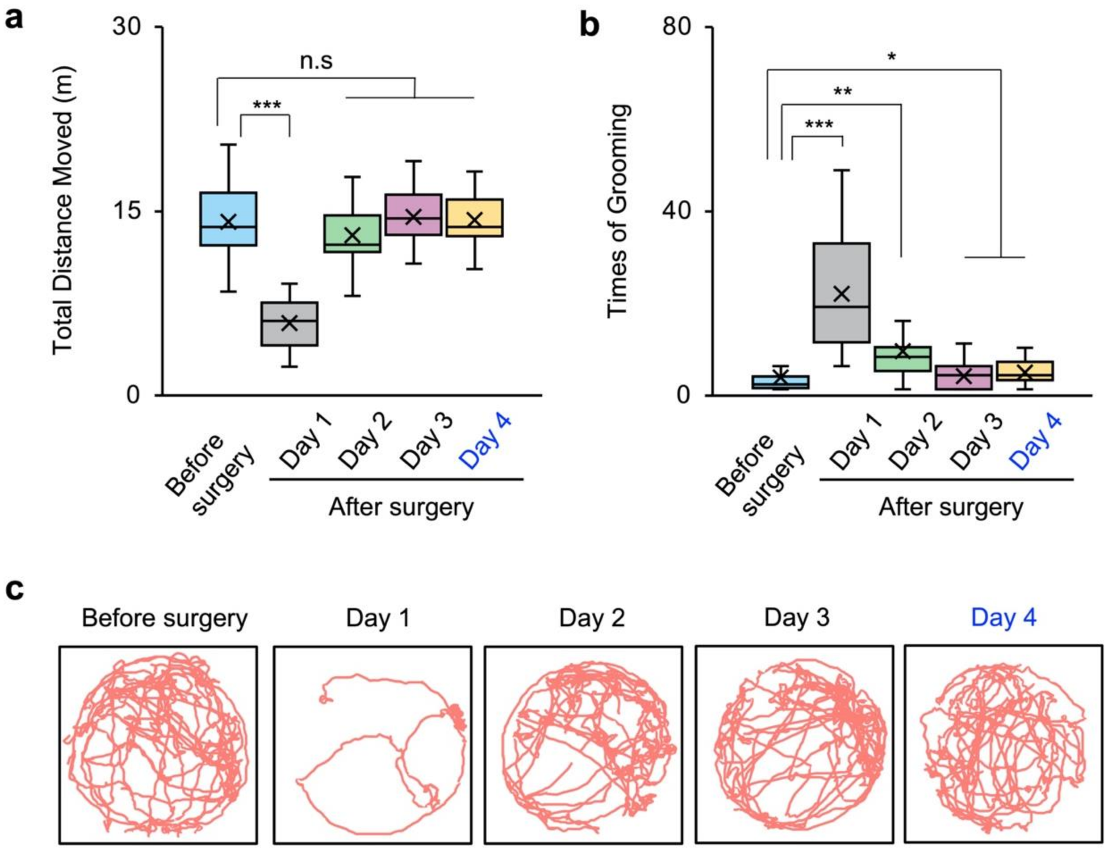

Figure S32. (**a–b**) The effect of surgery on motor activity and grooming times of mice (*n* = 8 mice). The box and whisker plots represent median values (horizontal bars), 25th to 75th percentiles (box edges) and minimum to maximum values (whiskers). Significance was determined by two-tailed unpaired Student's *t*-test (*$p < 0.05$, **$p < 0.01$, ***$p < 0.001$ and n.s., no significance). (**c**) The motion tracks of mice in 5 min in an empty circular arena.

Long-term caffeine intake. The experiments illustrated in **Figure S33a** were performed to study the effect of long-time high-dose caffeine injection on the brain function. **Figure S33b–i** display the results of two other groups, which coincide with those of Group 1 shown in **Figure 4g–j**. The motion tracks of mice (group 1-3) before and after 9 days high-dose caffeine injection or saline in 5 min in an empty circular arena were displayed in **Figure S34**.

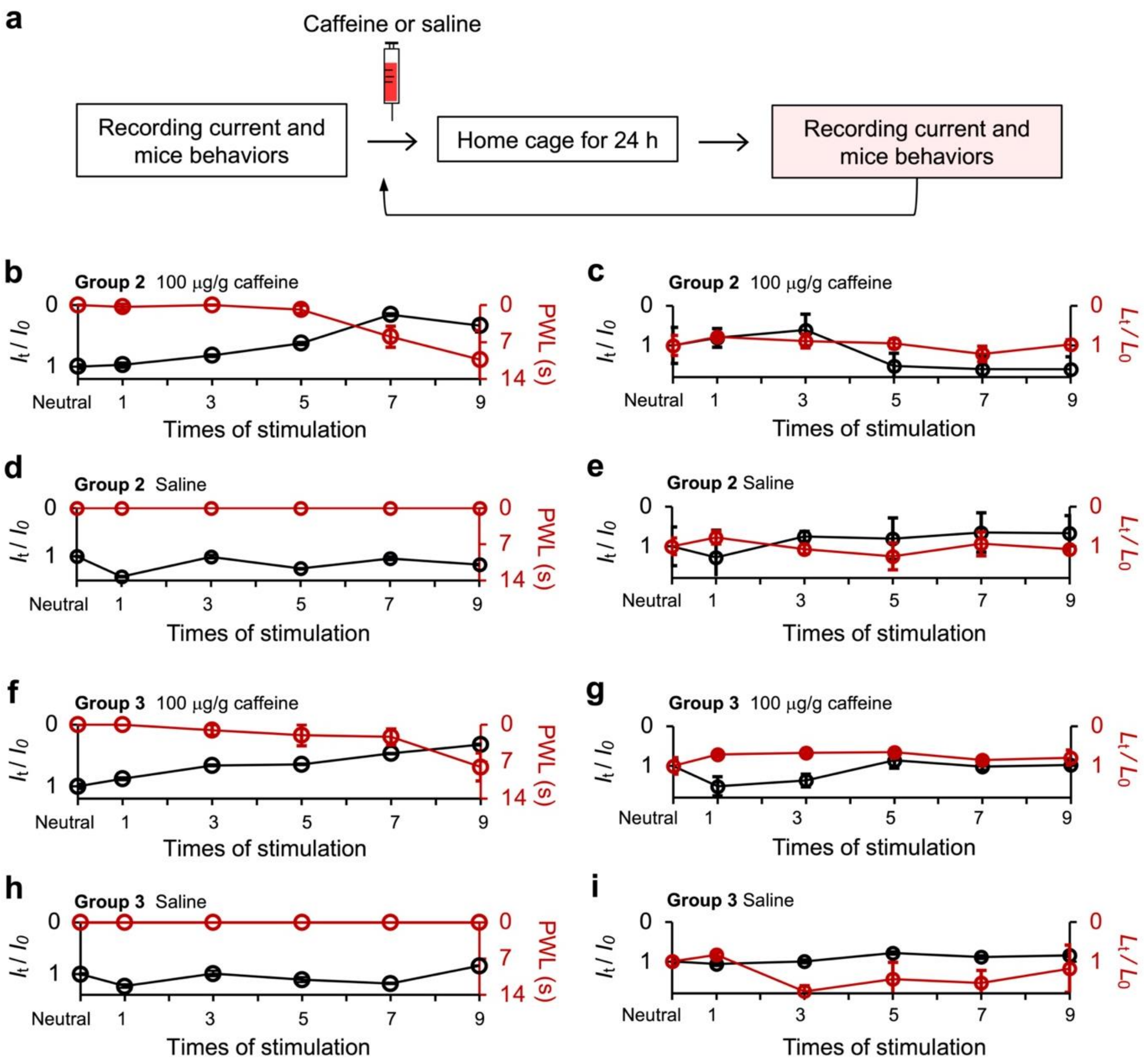

Figure S33. (**a**) The procedure of studying the effect of long-time high-dose caffeine injection on the function of S1 and M1. High-dose caffeine is injected daily for consecutive 9 days. Prior to caffeine injection, the current is measured and behaviors of mice are monitored. (**b–i**) The effect of high-dose caffeine (**b**, **c**, **f**, **g**) and saline (**d**, **e**, **h**, **i**) on I_t/I_0 measured with tCFE

in S1 (**b**, **d**, **f**, **h**) and M1 (**c**, **e**, **g**, **i**), on the PWL of mice in response to hindpaw mechanical force (**b**, **d**, **f**, **h**) and on L_t/L_0 (**c**, **e**, **g**, **i**). Both current and behavior of mice were measured right before caffeine or saline injection. Data are expressed as mean ± s.d.

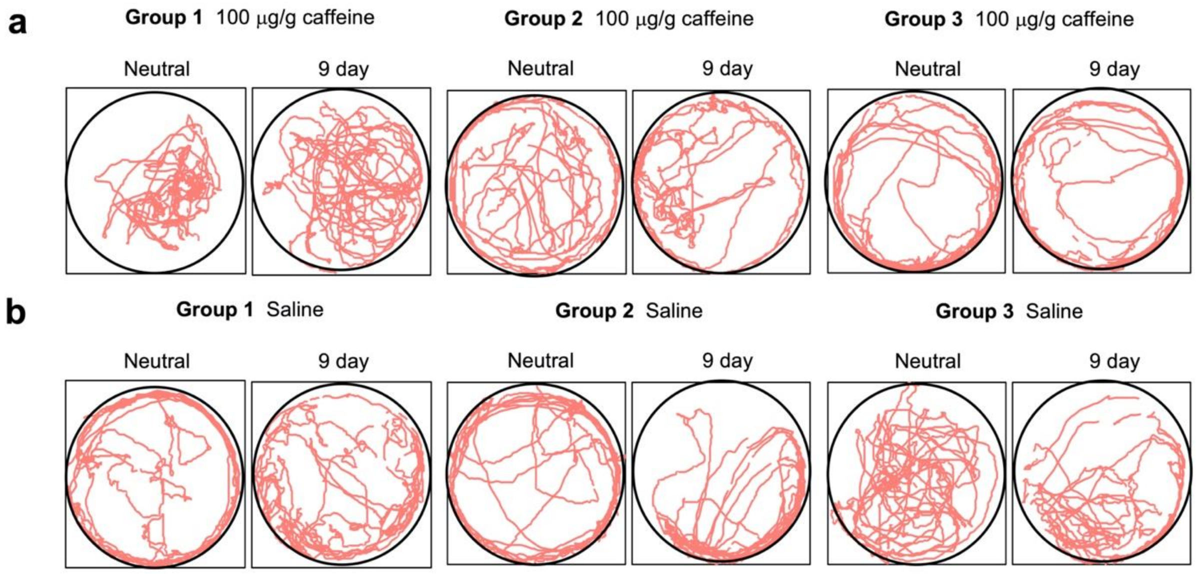

Figure S34. The motion tracks of mice (group 1-3) before and after 9 days high-dose caffeine injection (**a**) or saline (**b**) in 5 min in an empty circular arena.

Nissl and TUNEL staining. The neuronal damage of brain caused by consecutive high-dose caffeine injection was studied by Nissl and TUNEL staining.

Preparation of sections. Firstly, high-dose caffeine or saline (control experiment) was injected to the abdominal cavity of mice once per day for 9 days. Then, sections were prepared according to the approach described in **S5. Histology and immunohistochemistry.**

Nissl staining. Sections were stained with Nissl staining solution and mounted with the neutral balsam and observed under an inverted optical microscope (cx21, Nikon). The number of Nissl staining cells (*n*) was counted with Image J.

TUNEL staining. TUNEL staining was used to identify and quantify apoptotic neuronal cells. Firstly, the frozen sections were twice rinsed with 1× PBS for 5 min. Then, the sections were stained using a C1089 one step TUNEL apoptosis assay kit. Finally, the sections were mounted with the commercial antifade medium containing DAPI. Confocal fluorescence images were captured on a high-resolution microscope (IX83-FV3000-OSR, Olympus). The ratio between TUNEL-positive cells and number of cells stained with DAPI (defined as the percentage of TUNEL-positive cell) was analyzed using Image J.

Nissl and TUNEL staining tests showed that consecutive high-dose caffeine injection would cause neuronal damage in S1 (**Figures S35–36**). The number of Nissl staining cells in S1 of mice after consecutive injection of high-dose caffeine was significantly less than control groups (consecutive injection of saline). In addition, the percentage of TUNEL-positive cell was increased in S1 of mice after consecutive injection of high-dose caffeine. The results indicate that the low brain oxygen level induced by the strong inhibition effect of caffeine can indeed cause neuronal damage in S1, which matches well with the decrease of I_t/I_0 in S1 and increase of PWL (**Figure 4g** and **Figure S33b, f**). Nissl and TUNEL staining tests also show that the number of Nissl staining cells and the percentage of TUNEL-positive cells in M1 of mice after consecutive injection of high-dose caffeine are comparable to control groups, suggesting that a slight inhibition effect of caffeine on M1 will not cause neuronal damage in M1.

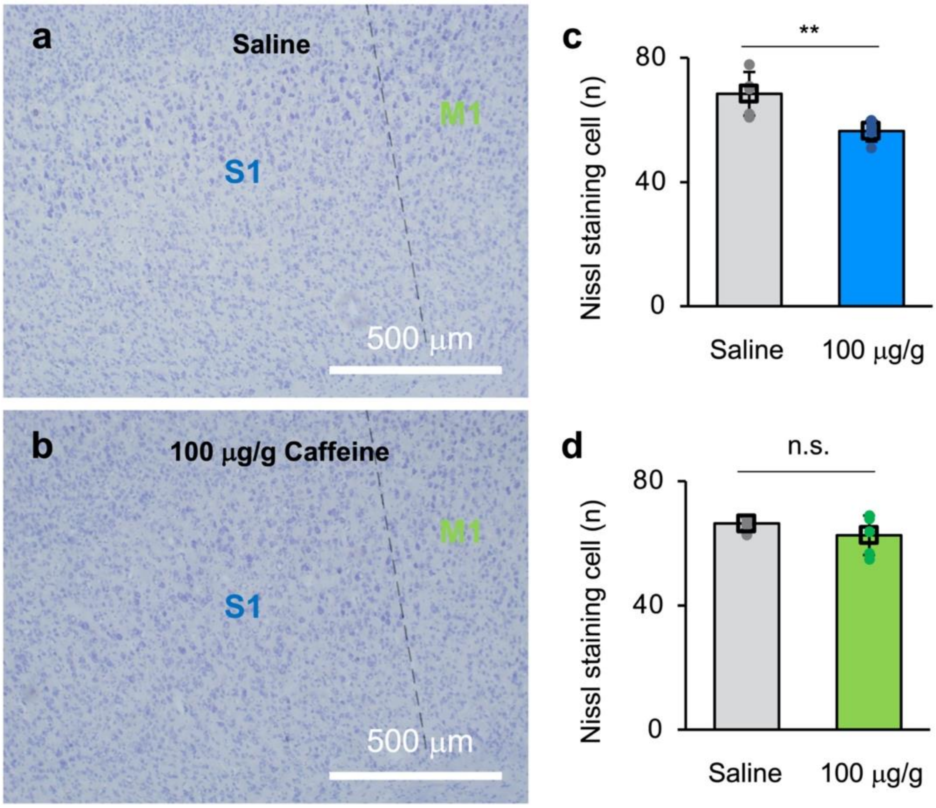

Figure S35. (**a**–**b**) Optical images of Nissl staining of S1 (**a**) and M1 (**b**) obtained with mice after consecutive injection of saline and high-dose caffeine for 9 days. (**c**–**d**) Statistical analysis of the number of Nissl straining cells (n = 5 mice). Data are expressed as mean ± s.d. Significance was determined by two-tailed unpaired Student's t-test (**$p < 0.01$ and n.s., no significance).

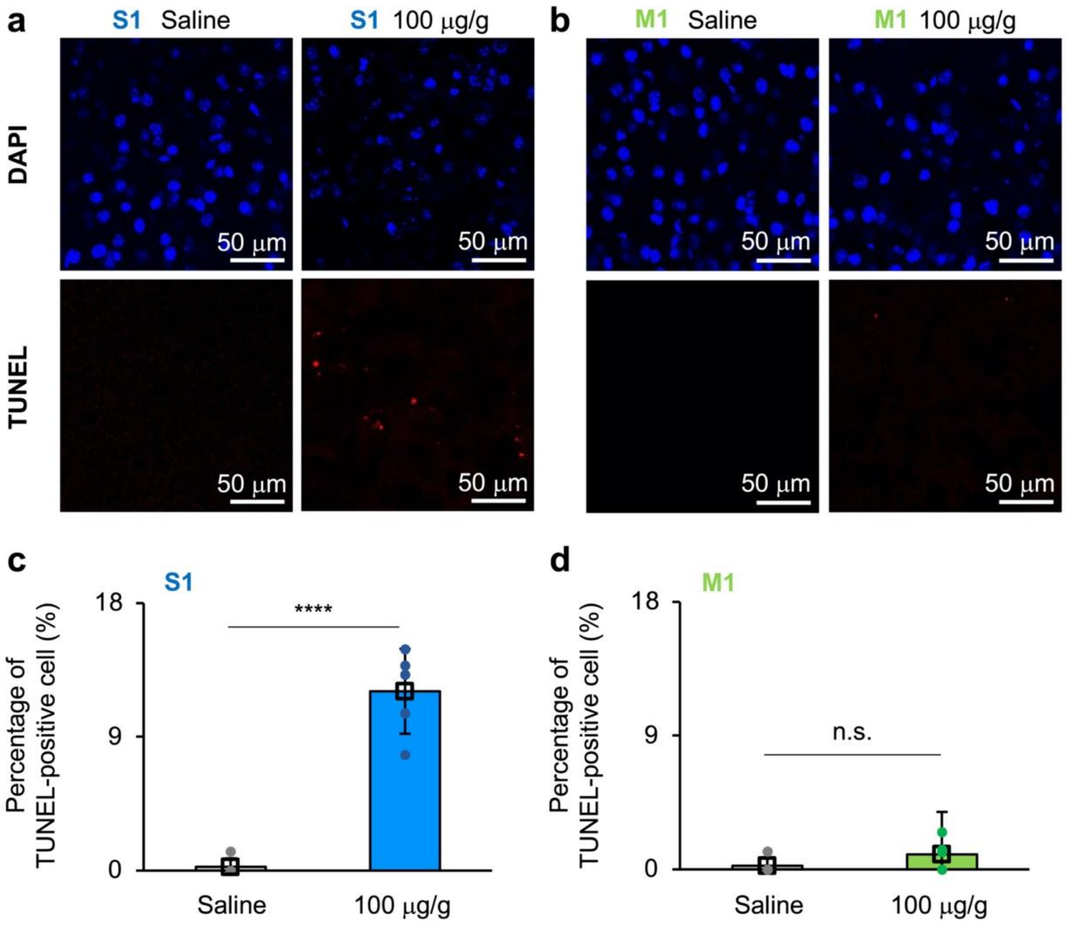

Figure S36. (**a**–**b**) Confocal fluorescence images of DAPI and TUNEL staining of S1 (**a**) and M1 (**b**) obtained with mice after consecutive injection of saline and high-dose caffeine for 9 days. (**c**–**d**) Statistical of the percentage TUNEL-positive cells (*n* = 5 mice). Data are expressed as mean ± s.d. Significance was determined by two-tailed unpaired Student's *t*-test (*****p* < 0.0001 and n.s., no significance).

Movie

Movie 1. Video of convulsion induced by high-dose caffeine stimulation.

Movie 2. Freely moving mouse with custom flexible potentiostat.

Reference

[S1] L. Zhou, H. Hou, H. Wei, L. Yao, L. Sun, P. Yu, B. Su, L. Mao, *Anal. Chem.* **2019**, *91*, 3645.

[S2] A. Pabbathi, S. Patra, A. Samanta, *ChemPhysChem* **2013**, *14*, 2441.

[S3] A. Walcarius, E. Sibottier, M. Etienne, J. Ghanbaja, *Nat. Mater.* **2007**, *6*, 602.

[S4] X. R. Li, L. Zhou, J. L. Ding, L. Sun, B. Su, *ChemElectroChem* **2020**, *7*, 2081.

[S5] L. Zhou, C. Cheng, X. Li, J. Ding, Q. Liu, B. Su, *Anal. Chem.* **2020**, *92*, 3844.

[S6] Q. Wei, T. Becherer, S. Angioletti-Uberti, J. Dzubiella, C. Wischke, A. T. Neffe, A. Lendlein, M. Ballauff, R. Haag, *Angew. Chem., Int. Ed.* **2014**, *53*, 8004.

[S7] J. J. Gooding, S. Ciampi, *Chem. Soc. Rev.* **2011**, *40*, 2704.

[S8] J. Sabate Del Rio, O. Y. F. Henry, P. Jolly, D. E. Ingber, *Nat. Nanotechnol.* **2019**, *14*, 1143.

[S9] Z. Teng, G. Zheng, Y. Dou, W. Li, C. Mou, X. Zhang, A. M. Asiri, D. Zhao, *Angew. Chem., Int. Ed.* **2012**, *51*, 2173.

[S10] L. Wang, S. Xie, Z. Wang, F. Liu, Y. Yang, C. Tang, X. Wu, P. Liu, Y. Li, H. Saiyin, S. Zheng, X. Sun, F. Xu, H. Yu, H. Peng, *Nat. Biomed. Eng.* **2019**, *3*, 1.

[S11] C. Chatard, A. Sabac, L. Moreno-Velasquez, A. Meiller, S. Marinesco, *ACS Cent. Sci.* **2018**, *4*, 1751.
